# Integrating Host Plant Availability into Ensemble Models Reveals Divergent Responses of Three Endangered Papilionid Butterflies to Climate Change in China

**DOI:** 10.3390/biology15151232

**Published:** 2026-07-24

**Authors:** Ze Lan, Guangfu Zhang

**Affiliations:** Jiangsu Key Laboratory of Biodiversity and Biotechnology, School of Life Sciences, Nanjing Normal University, 1 Wenyuan Road, Nanjing 210023, China; 241202137@njnu.edu.cn

**Keywords:** species distribution models, Papilionidae, climate change, habitat suitability, spatial fragmentation

## Abstract

Climate change is altering the potential distribution of many butterfly species, but climatic suitability alone may be insufficient to sustain the long-term viability of host-dependent butterflies. For papilionid butterflies, larval feeding and development depend on specific host plants; however, the extent to which host plant availability modifies projected suitable habitats under climate change remains unclear. Here, we first selected three threatened papilionids in China: *Luehdorfia chinensis* Leech, *Teinopalpus imperialis* Hope, and *Troides aeacus* C. & R. Felder (Lepidoptera: Papilionidae). Then we constructed two sets of ensemble distribution models: an environment-only model (Env-only) and a host-inclusive model (Env+Host), and compared the resulting projections for each species under current and future climate scenarios (the 2050s, 2070s, and 2090s under SSP1–2.6, SSP2–4.5, and SSP5–8.5). Landscape pattern metrics were further employed to assess changes in the spatial structure of suitable habitats. In the Env-only models, precipitation-related variables were identified as the primary factors constraining the potential distributions of all three species. After it was incorporated, host plant availability became the most important predictor for *L. chinensis* (41.54%) and *T. aeacus* (51.53%). In contrast, precipitation of the warmest quarter remained the dominant predictor for *T. imperialis* (36.00%). Compared with the Env-only models, the Env+Host models forecast smaller suitable habitats for all three species under all scenarios. *T. imperialis* exhibited the largest host-related contraction under SSP5–8.5 2090s (68.44%). Additionally, landscape pattern metrics indicated increased habitat fragmentation for *L. chinensis* and *T. imperialis*, but enhanced spatial continuity for *T. aeacus*. Overall, the three species have pronounced interspecific differences in their responses to the incorporation of host plant availability. These findings suggest that biotic factors should be incorporated into suitable habitat assessments for threatened papilionids in China. Accordingly, conservation strategies should be tailored to their host specificity, climate-driven distribution, and habitat requirements.

## Simple Summary

Climate change is reshaping the distribution patterns of many butterflies, but climatic suitability alone may not ensure their persistence. Papilionid larvae depend on specific host plants for feeding and development. In this study, we examined the potential distributions of three threatened papilionids in China: *Luehdorfia chinensis* Leech, *Teinopalpus imperialis* Hope, and *Troides aeacus* C. & R. Felder (Lepidoptera: Papilionidae). We compared predictions from environmental conditions alone with those from inclusive larval host plant availability. Incorporating host plants reduced suitable habitats for all three species. Host plants strongly influenced the predicted distributions of *L. chinensis* (41.54%) and *T. aeacus* (51.53%), whereas *T. imperialis* remained primarily constrained by precipitation of the warmest quarter (36.00%). Compared with the Env-only models, the Env+Host models projected smaller suitable habitats for all papilionids species under all scenarios. Four landscape pattern metrics further indicated increased habitat fragmentation for *L. chinensis* and *T. imperialis*, but enhanced spatial continuity for *T. aeacus*. These findings indicate that suitable habitat assessments for threatened papilionids should consider environmental conditions as well as host plants. Moreover, conservation strategies should account for host specificity, climatic constraints, and the spatial structure of suitable habitats.

## 1. Introduction

Butterflies are an important component of insect diversity, with approximately 19,500 extant species currently recognized worldwide, and they play key roles in pollination, food-web organization, and the maintenance of ecosystem functioning [1,2]. Owing to their relatively short life cycles and high sensitivity to variation in temperature and precipitation, butterflies are widely used as indicator taxa for assessing the effects of climate change on insect diversity and distribution patterns [3]. Previous studies have shown that climate change can alter butterfly phenology, developmental rates, thermal tolerance, behavior, and voltinism, thereby affecting population dynamics, community composition, and long-term persistence [3,4]. Spatially, climate warming has caused range expansions or contractions in many butterfly species, poleward or upslope shifts in range boundaries, and community reorganization along latitudinal and elevational gradients [5,6,7]. Current research on butterfly responses to climate change generally follows two major approaches. One uses historical records, long-term monitoring data, and contemporary occurrence information to examine temporal changes in species distribution boundaries, occurrence frequency, and community composition [6,8]. The other applies species distribution models (SDMs), which integrate species occurrence records with environmental variables and have been widely used to assess climate-change impacts and predicting potential suitable habitats under current and future climate scenarios [9,10].

The family Papilionidae represents a distinctive lineage of extant butterflies within Papilionoidea, comprising approximately 600 species worldwide and accounting for nearly 3% of the global butterfly diversity [1,11]. Although this proportion is relatively modest, papilionid butterflies have long attracted sustained attention in ecology, evolutionary biology, and conservation biology because of their distinctive morphology, close host plant associations, complex evolutionary history, and high conservation value [1,11]. As holometabolous insects, papilionid butterflies pass through egg, larval, pupal, and adult stages, each with distinct ecological roles. Adults feed mainly on nectar and function as pollinators of certain flowering plants; larvae, as herbivorous insects, participate directly in plant–insect interactions; and eggs, larvae, pupae, and adults also serve as important resources for predators and parasitoids, thereby contributing to food-web structure [2,12]. More importantly, many papilionid species exhibit marked larval host plant specialization, and their larvae often feed on plants from a single family or a few closely related lineages [13,14]. These host plants are usually concentrated within a relatively limited set of plant lineages, including Aristolochiaceae, Magnoliaceae, Rutaceae, and other related groups [1,15].

The high degree of larval host specialization in Papilionidae suggests that papilionid responses to climate change may be constrained not only directly by climatic conditions such as temperature and precipitation, but also indirectly through shifts in the distributions of larval hosts. Traditional SDMs typically use abiotic variables, such as climate, topography, and anthropogenic disturbance, as the main predictors [9]. However, growing evidence indicates that neglecting interspecific interactions may lead to biased estimates of species’ realized distributions [16,17,18], especially at large spatial scales. This underestimated interspecific interaction may be particularly important for host-specialist or oligophagous insects, because host plant availability can directly influence adult oviposition, larval feeding, population colonization, and long-term persistence [19,20,21]. For such papilionids, an area may be climatically suitable yet still fail to constitute truly available habitat if larval host plants are absent. Previous studies have shown that the occurrence of *Teinopalpus aureus* in montane forests of southern China is jointly constrained by forest quality and the availability of magnoliaceous larval host plants, whereas the rapid northward expansion of *Papilio cresphontes* in northeastern North America has been driven together by climate warming and local host plant availability [20,22]. These findings imply that host plant availability should be considered a biologically meaningful constraint when modelling host-dependent butterflies, particularly species with narrow distributions or high conservation concern. At broader spatial scales, however, climatic conditions still remain the dominant constraints on species distributions [18,23,24]. In addition, incomplete host plant distribution data, uncertainty in future host plant projections, and correlations between host-related and climatic variables may all increase the complexity of model interpretation [7,19]. Therefore, for threatened papilionid butterflies with pronounced host plant specificity, it remains unclear whether, and to what extent, incorporating host plant availability substantially changes predictions of suitable habitat relative to models based on abiotic environmental variables alone.

China is a major center of butterfly diversity in East Asia. Its complex topography, pronounced climatic gradients, and diverse vegetation types provide butterfly assemblages with highly heterogeneous habitats and food resources [2,25]. At the regional scale, central and southern China includes several mountainous and subtropical regions with high butterfly richness, such as the Qinling Mountains in central China [26] and the Hengduan Mountains in southwestern China [27]. Papilionidae in China comprises 371 species (including subspecies-level taxa), approximately 60% of the 600 known papilionid species worldwide [28]. In the 2021 List of National Key Protected Wild Animals, 24 butterfly species were included under national protection in China. Among them, 18 belonged to Papilionidae, accounting for 75% of all nationally protected butterfly species [29]. Nevertheless, studies on the suitable habitats of threatened papilionid butterflies in China remain limited [30], and it is unclear how host plant availability may affect predictions or whether this effect differs among species.

Compared with previous studies that have often focused on single butterfly species or primarily emphasized abiotic environmental suitability, the three focal species provide a comparative system for evaluating species-specific responses to host-plant incorporation. All three species are threatened papilionids [31,32,33,34,35] and are listed as Class II nationally protected wildlife in China [29]. In addition, their larvae depend on specific host plants, consistent with the pronounced host specialization of Papilionidae [13,14]. However, these papilionids differ markedly in host plant breadth, geographic range, and the climatic–topographic backgrounds of their distributions. Specifically, *Luehdorfia chinensis* Leech is mainly associated with Aristolochiaceae hosts, *Teinopalpus imperialis* Hope is associated with *Daphne* and *Yulania* host plants, whereas *Troides aeacus* C. & R. Felder uses multiple *Aristolochia* species (Table S2) [36]. They differ in geographical distribution. *L. chinensis* is mainly distributed in central-eastern to southeastern China [35,37], *T. imperialis* occurs more locally in montane southwestern China [31,38], and *T. aeacus* has a broader distribution across southern China [31,39,40]. Therefore, selecting these three species allows us to compare whether host-plant availability affects suitable-habitat projections and whether such effects differ in magnitude among papilionids.

Hence, this study compares models with and without larval host plant availability to evaluate how host plant factors affect the potential suitable habitats of three threatened papilionid butterflies in China under current and future climate scenarios. Specifically, this study addresses three questions: (1) How does the incorporation of host plant availability affect the relative contributions of environmental variables and host plant availability in distribution models for each of the three papilionid species? (2) How does each papilionid species change in extent and pattern of suitable habitat when host plant availability is incorporated? (3) How does incorporating host plant availability further alter suitable habitat quality, as proxied by landscape pattern metrics? This study aims to reveal interspecific differences in responses to climate change and host-related constraints among these threatened papilionids, thereby informing climate-adaptive strategies for their conservation and habitat management in China.

## 2. Materials and Methods

### 2.1. Study Species and Occurrence Records

In this study, we first selected three swallowtail butterflies, namely *L. chinensis*, *T. imperialis*, and *T. aeacus*. Then we compiled the occurrence records for each of the butterflies and its corresponding larval host plants. For each papilionid, the occurrence records of its corresponding host plants were used to generate a host-plant availability variable, which was subsequently incorporated into butterfly SDMs as a biotic predictor. The larval host plant checklists of the three butterfly species, together with the number of occurrence records for both butterflies and hosts, are provided in Tables S1 and S2.

Distribution records of these butterfly species were mainly derived from the Global Biodiversity Information Facility (GBIF), the National Specimen Information Infrastructure (NSII), as well as published literature on taxonomy, fauna, and distribution. Specifically, occurrence data for *L. chinensis* were based primarily on the dataset compiled by Lan and Zhang [35], and were further supplemented and cross-validated with GBIF occurrences (https://doi.org/10.15468/dl.ftk34k, accessed on 21 May 2026), NSII specimen records, and relevant literature [37,41]. The records for *T. imperialis* were systematically compiled from GBIF occurrence downloads (https://doi.org/10.15468/dl.p5axnx, accessed on 21 May 2026), NSII specimen records, and relevant distributional literature [31,38]. The occurrences for *T. aeacus* were based on an existing compilation of its distribution in China [40], and were further integrated with GBIF occurrence downloads (https://doi.org/10.15468/dl.yxp9fx, accessed on 21 May 2026), NSII specimen records, and published literature [31,39]. To ensure data quality, records obtained from different sources were cross-checked for consistency in species identity, locality descriptions, and geographic coordinates. All records were subsequently subjected to the same standardized quality-control and spatial-filtering procedures described below.

Information on larval host plants was obtained mainly from relevant published literature on the life history, larval feeding, and host use of the three butterfly species, and supplemented with the HOSTS database of the Natural History Museum, London [36]. These larval host plants included *Asarum sieboldii* Miq. and *A. forbesii* Maxim. for *L. chinensis*; *Daphne bholua* Buch.-Ham. ex D. Don and *Yulania campbellii* (Hook. f. & Thomson) D. L. Fu for *T. imperialis*; and ten species of Aristolochiaceae for *T. aeacus*. Then the larval host plant lists of the three butterfly species were determined, and their scientific names were checked for synonyms and standardized to accepted names. Only plant records with explicit evidence of larval feeding or host use were retained; records based solely on adult flower visitation, or lacking evidence of larval feeding, were excluded. After this screening, two host plant species were retained for *L. chinensis*; two were retained for *T. imperialis*; and six were retained for *T. aeacus*, namely *Aristolochia debilis* Siebold & Zucc., *A. kaempferi* Willd., *A. zollingeriana* Miq., *A. cucurbitifolia* Hayata, *A. liukiuensis* Hatus., and *A. griffithii* Hook. f. & Thomson ex Duch.

Host plant occurrences were collated from three main sources. The first source consisted of field investigation records, namely distributional records of wild host plant populations in eastern China, including Anhui, Jiangsu, Jiangxi, and Zhejiang provinces over the past decade. The second was from online databases and image-based records, including host plant records from NSII, GBIF, and the Plant Photo Bank of China (PPBC). In addition, these data were appropriately supplemented by recent research literature records. The third source consisted of supplementary literature records derived from taxonomic studies, floras, floristic surveys, and published studies on plant distributions; these records were used to supplement and cross-validate host plant occurrence data.

To reduce the effects of coordinate errors, duplicate records, and sampling bias on model outputs, raw occurrence points of both butterflies and host plants were subjected to a unified quality-control procedure. First, records lacking coordinates, containing obvious coordinate errors, showing coordinate uncertainty clearly incompatible with a 1 km spatial resolution, or not reliably georeferenceable from locality descriptions were removed. Second, exact duplicate records, records with obviously erroneous terrestrial or marine coordinates, and records from cultivated, introduced, ornamental, or other non-natural habitats were also excluded.

**Figure 1:**
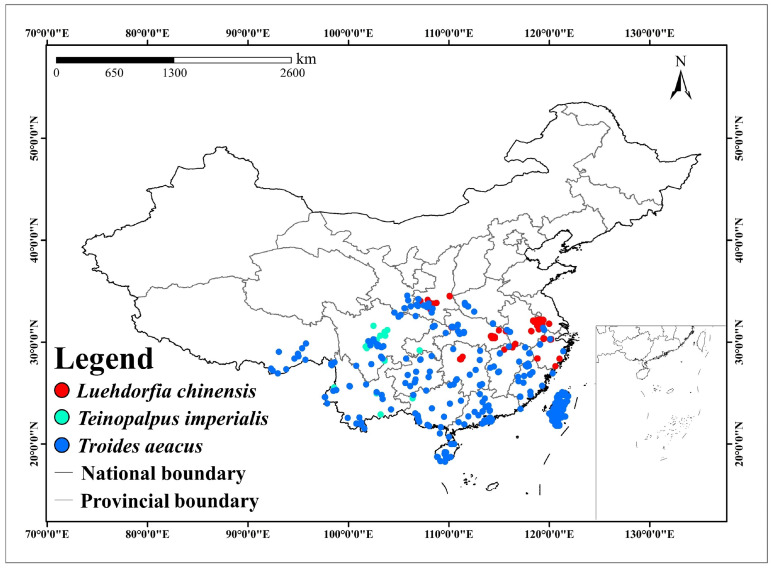
Spatial distribution of occurrence records for three papilionid butterflies.

Third, for records with detailed locality descriptions but without geographic coordinates, locality names were parsed and their coordinates were assigned following standard georeferencing procedures for biodiversity occurrence data [42]. These positions were then verified in Google Earth. All coordinates were standardized to the WGS 84 geographic coordinate system. Before SDM construction, the cleaned occurrence records were further spatially thinned in SDMtoolbox 2.0 to reduce spatial autocorrelation and sampling bias [43]. Thinning was conducted at a 1 km spatial resolution, such that only one occurrence record was retained within each 1 km grid cell. Through this standardized process of record screening, georeferencing, coordinate verification, coordinate-system standardization, and spatial thinning, the resulting number of occurrence records was 150 for *L. chinensis*, 20 for *T. imperialis*, and 441 for *T. aeacus* in the final model, respectively (Figure 1; Table S3). The differences in occurrence points mainly reflected the actual availability of reliable records for each species after standardized quality control. Because these three butterfly species are modelled separately, the species with more points (i.e., *T. aeacus*) will not affect the model calibration of species with fewer points (i.e., *T. imperialis*).

### 2.2. Abiotic Variables

Candidate abiotic variables comprised 19 bioclimatic, three topographic, and one anthropogenic disturbance variable. The 19 bioclimatic variables were obtained from WorldClim v2.1 at a spatial resolution of 30 arc-seconds (~1 km) [44]. Topographic predictors were derived from a digital elevation model (DEM). Specifically, the SRTM 1 Arc-Second Global DEM was used, from which elevation, slope, and aspect were extracted [45]. Anthropogenic influence was represented using the global Human Influence Index (HI) dataset released by the NASA Socioeconomic Data and Applications Center (SEDAC). This dataset integrates population density, built-up areas, nighttime lights, land-use/land-cover data, and human access features, including roads, railways, coastlines, and navigable rivers. It comprises nine indicator layers and is used to characterize the intensity of human disturbance across the study area [46].

Future climate projections were based on the same data framework and spatial resolution as the current climate data. Current climate variables were obtained from WorldClim v2.1, whereas future climate projections were derived from the CMIP6 downscaled datasets provided by WorldClim. The BCC-CSM2-MR global climate model was selected, and three future time slices were considered: 2041–2060, 2061–2080, and 2081–2100, hereafter referred to as the 2050s, 2070s, and 2090s, respectively. Three Shared Socioeconomic Pathway scenarios, SSP1–2.6, SSP2–4.5, and SSP5–8.5, were selected to represent future climate change under low-, intermediate-, and high-emission conditions, respectively [47,48,49].

The BCC-CSM2-MR model has been widely used in climate-change projection and species distribution studies [50,51,52]. Compared with earlier BCC models, BCC-CSM2-MR includes improvements in physical parameterizations and thus shows better performance in simulating major climate variables, particularly in East Asia [53]. In addition, previous evaluations have indicated that BCC-CSM2-MR performs relatively well in simulating precipitation patterns in China and the East Asian summer monsoon [54]. Furthermore, it may be convenient to compare the results from other papilionid butterflies with this study when using the same climate model.

To avoid model overfitting, parameter instability, and interpretive bias arising from multicollinearity, pairwise Pearson correlation coefficients (r) were calculated for all candidate abiotic variables, and correlation-based screening was conducted in SDMtoolbox 2.0. Variable pairs with |*r*| > 0.8 were regarded as highly correlated [55]. For each such pair, the variable with the higher contribution in preliminary models was retained in the final predictor set, whereas the other was excluded [43,56]. Specifically, the retained variables for *L. chinensis* were aspect, Bio02, Bio03, Bio04, Bio09, Bio15, Bio17, elevation, human influence index, and slope; for *T. imperialis* were aspect, Bio02, Bio03, Bio07, Bio09, Bio10, Bio14, Bio15, Bio18, human influence index, and slope; for *T. aeacus* were aspect, Bio03, Bio06, Bio07, Bio12, Bio15, elevation, human influence index, and slope (Figure S1). These environmental variables for the larval host plant models are summarized in Table S4.

### 2.3. Biotic Variables

Given that host plant availability is an important biotic factor constraining the distributions of monophagous or oligophagous butterflies, it was incorporated into butterfly SDMs as a biotic predictor representing the spatial availability of larval host resources. First, each host plant species associated with each butterfly species was modeled separately. The occurrence records, number of retained single models, and ensemble model performance of host plants are provided in Table S5. The procedures for occurrence data compilation, quality control, spatial thinning, abiotic variable selection, model construction, and model evaluation were kept consistent with those used for butterfly SDMs in order to generate current potential suitability layers for each host plant species. Future host plant availability was not assumed to remain the same as current host plant availability. For each future period and each SSP scenario, habitat suitability was independently projected under each future period and SSP scenario using the corresponding future environmental variables. The resulting scenario-specific host suitability layers were then binarized and merged to generate the host availability layer. Accordingly, such a layer matched each of the nine climate scenarios (SSP1–2.6 in the 2050s, 2070s, and 2090s; SSP2–4.5 in the 2050s, 2070s, and 2090s; and SSP5–8.5 in the 2050s, 2070s, and 2090s).

To generate biotic predictor layers that could be used directly in subsequent analyses, the continuous suitability outputs of each host plant model were binarized using the maximum sum of sensitivity and specificity threshold (maxSSS). Grid cells with values greater than or equal to this threshold were classified as suitable (1), whereas those below the threshold were classified as unsuitable (0) [57,58,59]. For butterfly species with multiple host plants, the binary suitability layers of all corresponding host plants were overlaid at the raster level. If at least one host plant species was predicted to be suitable in a given grid cell, that cell was assigned a value of 1; otherwise, it was assigned a value of 0. Continuous suitability does not directly indicate host abundance, whereas richness and weighted indices require assumptions about host equivalence or species-specific weights. We therefore used a binary layer to indicate whether at least one potentially suitable larval host plant was available within each grid cell. This procedure generated a host plant availability layer for each butterfly species, which was then incorporated into the butterfly SDMs as a biotic predictor [17,19].

### 2.4. Model Calibration and Evaluation

To evaluate the effects of host availability on butterfly suitable areas, two sets of SDMs were constructed: (1) an environment-only model (Env-only), in which the selected abiotic variables, including climatic, topographic, and anthropogenic disturbance variables, were used as predictors; and (2) a host-inclusive model (Env+Host), in which the host plant availability layer was added to the Env-only model as a biotic predictor.

Prior to modelling, the common spatial extent in which all environmental rasters had valid values was defined as background extent. Subsequently, grid cells containing presence records were excluded from the background extent, and pseudo-absence points were randomly sampled from the remaining valid cells [60]. For each species, the number of pseudo-absence points was set to ten times the number of retained presence records. Presence records were coded as 1 and pseudo-absence records as 0. These data, together with the abiotic variables and host plant availability layers, were then imported into the biomod2 4.2.6.2 framework for model fitting and ensemble prediction [61].

Cross-validation was implemented using a spatial blocking strategy. First, the response data and environmental predictors were projected into a common planar coordinate system. The R 4.4.1 package blockCV 3.2.0 was then employed to determine block size according to the spatial autocorrelation range of the environmental rasters, and five-fold spatial block cross-validation was conducted [62,63,64]. Candidate block sizes ranging from 10 to 300 km were tested for each species. A block size of 150 km was finally selected because it allowed all three species to be divided into five spatial folds, and ensured that each testing fold contained presence records. In each cross-validation run, models were calibrated using four spatial folds and evaluated using the withheld fold [62,63]. To account for variability associated with pseudo-absence sampling and model calibration, the modelling procedure was repeated ten times for each species and each model type. In each repetition, pseudo-absence points were randomly re-sampled from the valid background cells, and five-fold spatial block cross-validation was applied.

The two model sets were fitted separately within the biomod2 4.2.6.2 framework. Candidate single-model algorithms included GLM, GAM, CTA, GBM, ANN, SRE, FDA, MARS, RF, and Maxent [61]. Model performance was evaluated using the area under the receiver operating characteristic curve (AUC) and the true skill statistic (TSS) [65]. Only single-model algorithms that simultaneously satisfied AUC > 0.8 and TSS > 0.7 were retained for ensemble prediction [66,67]. Because poorly performing models had already been excluded using the combined AUC and TSS thresholds, ensemble predictions were generated by assigning equal weights to all retained individual models [61,68,69,70].

### 2.5. Geospatial Analysis

To visualize changes in potential distribution patterns under different climate scenarios, ensemble predictions were mapped and spatially processed in ArcGIS 10.8, and raster calculations were performed using tools such as Map Algebra in Spatial Analyst.

Continuous suitability outputs were further reclassified into unsuitable, low-, moderate-, and high-suitability categories, with the species-specific maxSSS value used as the threshold separating unsuitable from suitable conditions. For butterflies, the total suitable area was defined as the sum of the low-, moderate-, and high-suitability areas, whereas for host plants it was defined as the sum of the moderate- and high-suitability areas [57,58,59]. Moreover, raster overlay analysis was performed on the binarized outputs of the two model types to compare changes in suitable areas between the Env+Host and Env-only models. The overlay order was set as “Env+Host minus Env-only”, and the resulting cells were classified into four categories: 0–0, unsuitable in both models, was defined as stable unsuitable area; 1–1, suitable in both models, was defined as stable suitable area; 0–1 indicated areas predicted to be suitable only by the Env-only model; and 1–0 indicated areas predicted to be suitable only by the Env+Host model. To assess the environmental transferability of future projections, we performed a Multivariate Environmental Similarity Surface (MESS) analysis following Elith et al. [71], using the predictor values from the model-calibration data as the reference set and the corresponding future predictor layers as the projection space. Grid cells with negative MESS values were identified as extrapolation-risk cells, and their percentage among all valid projection cells was calculated for each species, model type, future period, and SSP scenario.

### 2.6. Suitable Habitat Analysis

In addition to quantifying changes in the total suitable area, we further used landscape metrics to evaluate how host plant incorporation affected the spatial structure of suitable habitats for the three swallowtail butterflies. All landscape analyses were based on binary suitable-area rasters generated from ensemble models that had been calibrated and independently evaluated using five-fold spatial block cross-validation with ten repetitions. Accordingly, landscape pattern analysis was conducted using the binary suitable-area rasters derived from the Env-only and Env+Host models.

Herein, four landscape metrics were calculated using the R package landscapemetrics 2.2.1: NP, MPA, LPI, and SPLIT. NP (Number of Patches) refers to the number of suitable patches, indicating the degree to which suitable habitat was subdivided into separate patches. MPA (Mean Patch Area) refers to the mean area of all suitable patches, which can partly reflect the potential capacity of individual patches to support local populations. LPI (Largest Patch Index) refers to the percentage of the total suitable habitat area occupied by the largest suitable patch, indicating the dominance and integrity of the largest core habitat patch. In addition, SPLIT (Splitting Index) indicates the degree to which the landscape is subdivided into smaller patches; higher values indicate more dispersed and fragmented suitable habitat and stronger spatial separation among suitable patches. Suitable patches were identified using an 8-neighbour connectivity rule, such that grid cells sharing an edge or a corner were assigned to the same patch [72].

We attempted to use the difference in each landscape metric before and after host incorporation to quantify the effects of host information on patch structure and fragmentation:

$$\Delta Metric\left(\%\right)=\left(\frac{Metric_{Env+Host}-Metric_{Env\text{-}only}}{Metric_{Env\text{-}only}}\right)\times 100$$

where Δ*Metric* (%) represents the percentage change in each landscape metric after host incorporation; Metric*_Env+Host_* and Metric*_Env-only_* denote the metric values calculated from the host-inclusive and environment-only models, respectively.

## 3. Results

### 3.1. Effects of Host Availability on Model Performance

The individual algorithms varied for each of the three swallowtail butterflies in terms of AUC and TSS; however, each ensemble model exhibited high and relatively stable predictive accuracy relative to single models (Table 1 and Table 2). In the environment-only models (Env-only), the ensemble model AUC and TSS values were 0.980 and 0.930 for *L. chinensis*, 0.993 and 0.991 for *T. imperialis*, and 0.904 and 0.769 for *T. aeacus*, respectively, indicating that the ensemble models for all three papilionid butterflies achieved high predictive accuracy and stable predictive performance.

Furthermore, the comparison between the Env+Host and Env-only ensemble models revealed that these species differed in their responses to host plant availability.

For *L. chinensis*, after host plant availability was incorporated, the AUC of the ensemble model increased from 0.980 to 0.987, whereas the TSS decreased from 0.930 to 0.908. For *T. imperialis*, the AUC and TSS decreased from 0.993 and 0.991 to 0.952 and 0.883, respectively. For *T. aeacus*, the AUC increased from 0.904 to 0.949, whereas the TSS decreased slightly from 0.769 to 0.762. Overall, the incorporation of host plant availability did not consistently improve ensemble model performance across species or evaluation metrics.

### 3.2. Shifts in Key Predictors After Host Inclusion

In the Env-only models, the primary predictors were Bio17 for *L. chinensis* (54.88%), Bio18 for *T. imperialis* (34.51%), and Bio12 for *T. aeacus* (52.37%), respectively (Table 3). Overall, the variables with the highest contributions in the Env-only models were all precipitation-related bioclimatic variables, indicating that precipitation plays an important role in shaping the potential distributions of these butterflies.

In the Env+Host models, the relative contributions of the major predictors changed to varying degrees for all three species. Overall, for *L. chinensis* and *T. aeacus*, host plant availability became the highest-contributing predictor, with contribution rates of 41.54% and 51.53%, respectively; meanwhile, Bio17 and Bio12, which were the dominant predictors in the Env-only models, declined to second place, with contribution rates of 19.47% and 15.72%, respectively. By contrast, for *T. imperialis*, Bio18 remained the primary predictor after host plant availability was incorporated (36.00%), whereas host plant availability ranked among the top four predictors with a contribution rate of 11.31%.

### 3.3. Current Habitat Changes After Host Inclusion

Based on the overlay analysis of the Env+Host and Env-only models, the proportions of stable suitable habitat for *L. chinensis*, *T. imperialis*, and *T. aeacus* were 65.64%, 49.30%, and 93.93%, respectively (Table 4). The corresponding proportions of host-related expansion areas were 1.70%, 19.58%, and 1.44%, respectively; and the proportions of host-related contraction areas were 32.67%, 31.12%, and 4.63%, respectively. Overall, *T. aeacus* showed the highest proportion of stable suitable habitat, indicating a high consistency between the Env+Host and Env-only models. *T. imperialis* exhibited high proportions of both host-related expansion and contraction areas, suggesting the strongest spatial response to host plant availability among these species. By comparison, *L. chinensis* showed predominantly stable suitable habitat, along with some peripheral contraction.

**Figure 2:**
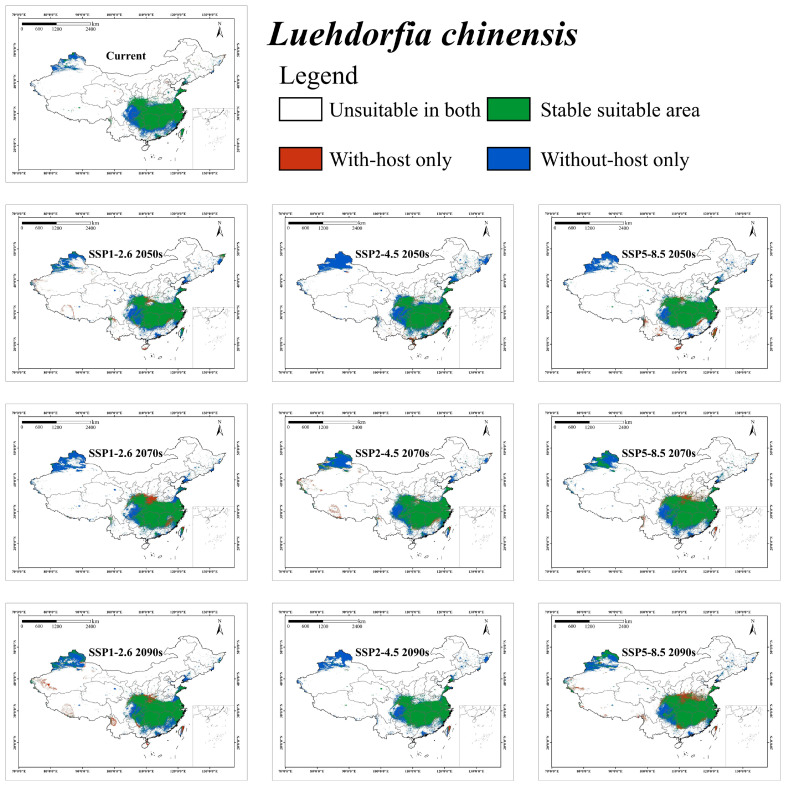
Overlap and differences in the potential suitable habitat of *Luehdorfia chinensis* between models with and without host plant availability under current and future climate scenarios. Black lines indicate national boundaries while grey lines indicate provincial boundaries.

**Figure 3:**
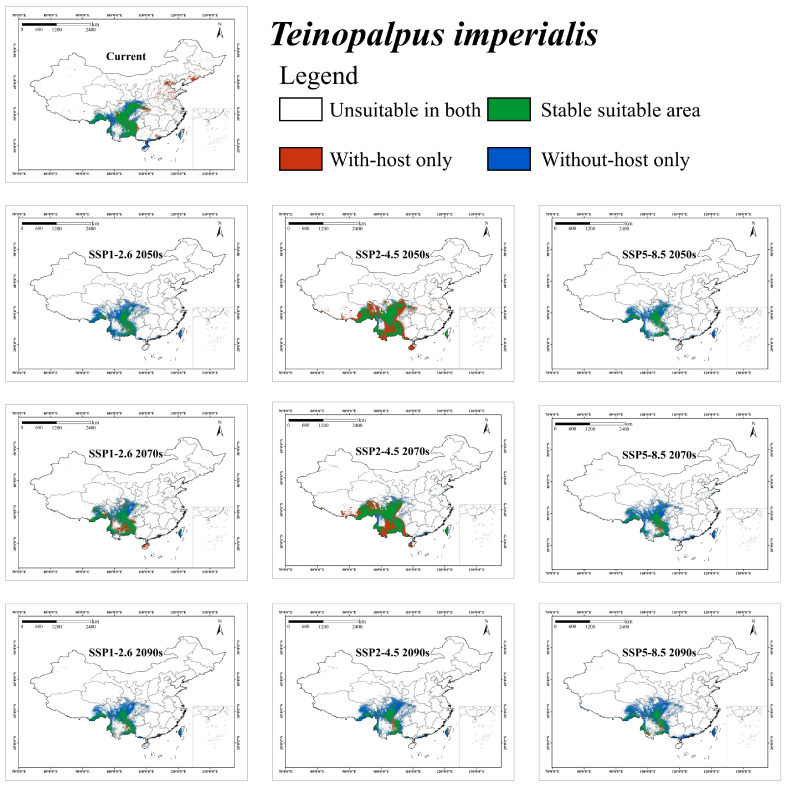
Overlap and differences in the potential suitable habitat of *Teinopalpus imperialis* between models with and without host plant availability under current and future climate scenarios. Black lines indicate national boundaries while grey lines indicate provincial boundaries.

**Figure 4:**
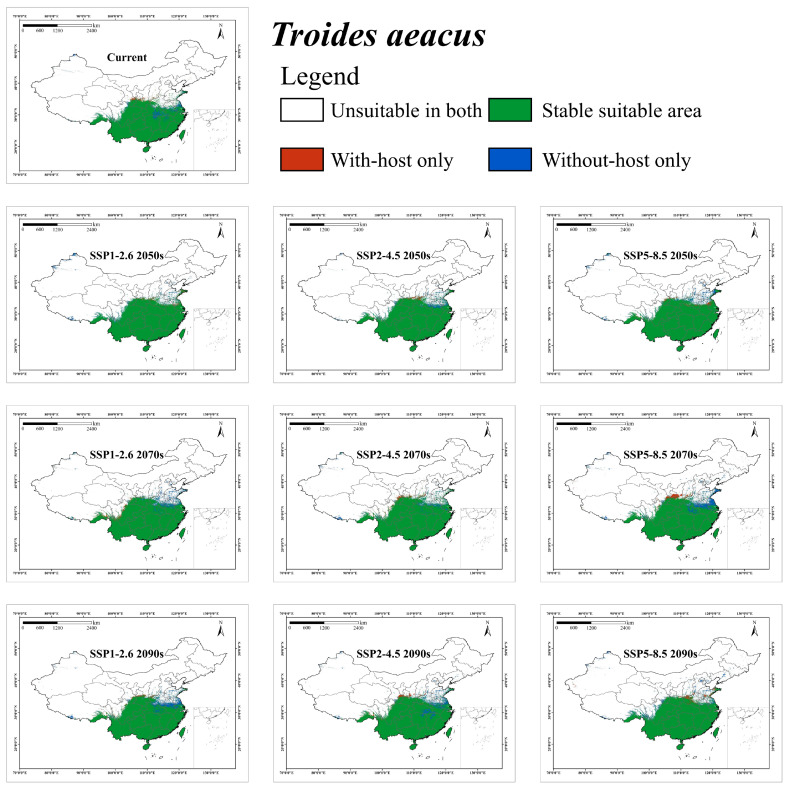
Overlap and differences in the potential suitable habitat of *Troides aeacus* between models with and without host plant availability under current and future climate scenarios. Black lines indicate national boundaries while grey lines indicate provincial boundaries.

The stable suitable habitat of *L. chinensis* was mainly distributed in central-eastern and southeastern China, including southern Anhui, eastern Chongqing, Fujian, northeastern Guizhou, Hubei, Hunan, Jiangsu, Jiangxi, southern Shaanxi, and Zhejiang. The contraction areas were mainly located around the periphery of the stable suitable habitat and within some isolated patch regions, primarily in northern Guangdong, northern Guangxi, western Guizhou, and eastern Sichuan (Figure 2). The stable suitable habitat of *T. imperialis* was mainly distributed in southwestern China, including northwestern Guangxi, western Guizhou, southwestern Sichuan, southeastern Xizang, and northeastern Yunnan. The contraction areas were mainly located in western Chongqing, northern Guizhou, and eastern Sichuan. The expansion areas were mainly scattered across southern Guangxi, central Guizhou, and southern Yunnan (Figure 3). The stable suitable habitat of *T. aeacus* was widely distributed across southern China, mainly in Chongqing, Fujian, Guangdong, Guangxi, Guizhou, Hainan, Hunan, Jiangxi, eastern Sichuan, and Yunnan, forming a relatively continuous core belt of suitable habitat. The contraction areas were small and mainly occurred in localized regions such as northern Anhui, eastern Hubei, and northern Hunan. The expansion areas were even more limited and were only sporadically distributed in Gansu and Shaanxi (Figure 4).

### 3.4. Future Habitat Stability and Reorganization

Across the nine future climate scenarios, the overlay analysis of the Env+Host and Env-only models was used to quantify the proportions of stable suitable habitat, host-related expansion areas, and host-related contraction areas for each species (Table 4). Overall, both *L. chinensis* and *T. aeacus* exhibited stable suitable habitat under future scenarios, with mean proportions of 61.27% and 92.42%, respectively. By contrast, *T. imperialis* showed a mean stable suitable habitat proportion of only 38.85%, with a mean contraction proportion of 49.41%, indicating that it had the strongest response to host plant availability among these species.

Furthermore, the mean proportions of host-related contraction and expansion areas for *L. chinensis* were 32.99% and 5.74%, respectively. For *T. aeacus*, the proportions of expansion and contraction areas were consistently low, at only 1.64% and 5.94%, respectively. These results indicate that incorporating host plant availability reduced the predicted suitable habitat areas of all three species, but the extent of reduction varied among species.

### 3.5. Changes in Fragmentation Patterns of Suitable Habitats

For *L. chinensis*, under the current climate scenario, NP in the Env+Host model decreased from 9931 to 5642 relative to the Env-only model, corresponding to a decrease of 43.19%. MPA and LPI decreased from 8.15 km^2^ and 24.42% to 2.44 km^2^ and 12.71%, respectively, representing decreases of 70.18% and 47.95%, whereas SPLIT increased from 16.77 to 61.86, increasing by 268.87% (Table 5; Table S6). Thus, except for NP, the other metrics consistently indicated an increase in habitat fragmentation under this scenario. Based on the mean values across future climate scenarios, NP increased from 5410.67 to 6334.89, corresponding to an increase of 17.08%; MPA and LPI decreased by 85.70% and 46.16%, respectively; and SPLIT increased by 248.58%. Together, these four metrics showed a consistent trend toward increased fragmentation in the future suitable habitat of *L. chinensis*.

For *T. imperialis*, NP increased from 70 to 11,631, corresponding to an increase of 16,515.71%; MPA and LPI decreased from 1356.11 km^2^ and 29.01% to 0.71 km^2^ and 5.26%, respectively, corresponding to decreases of 99.95% and 81.87%; and SPLIT increased from 11.88 to 353.38, corresponding to an increase of 2874.58%. The mean values across future climate scenarios showed a similar pattern, with NP and SPLIT increasing and MPA and LPI decreasing, suggesting that host incorporation consistently intensified habitat fragmentation for *T. imperialis* under both current and future climate scenarios.

For *T. aeacus*, under the current climate scenario, NP in the Env+Host model decreased from 5088 to 3858, corresponding to a decrease of 24.17%; MPA decreased from 15.86 km^2^ to 6.79 km^2^, corresponding to a decrease of 57.19%; LPI increased from 23.50% to 25.29%, corresponding to an increase of 7.62%; and SPLIT decreased from 18.09 to 15.61, corresponding to a decrease of 13.71%. Therefore, except for MPA, the other metrics consistently indicated enhanced habitat continuity under the current climate scenario. 

Based on the mean values across future climate scenarios, this trend became more evident. Specifically, NP fell by 70.81%, SPLIT dropped by 12.36%, meanwhile MPA and LPI rose by 11.75% and 6.84%, respectively. Collectively, these four metrics congruently showed a rising trend of habitat continuity in the future.

Overall, after incorporating host plant availability, both *L. chinensis* and *T. imperialis* showed increased fragmentation in suitable habitat under current and future scenarios, whereas *T. aeacus* showed increasing continuity across these scenarios.

## 4. Discussion

### 4.1. Model Evaluation and Variable Contribution

In the Env-only models, the retained individual algorithms differed among the three endangered papilionid butterflies according to the model selection criteria (AUC > 0.8 and TSS > 0.7). There were seven, six, and eight individual models retained in the final ensemble modelling for *L. chinensis*, *T. imperialis*, and *T. aeacus*, respectively (Table 1 and Table 2), suggesting species-specific differences in model fitting. Overall, compared with single models, the Env-only ensemble models showed better performance in terms of AUC and TSS, indicating that ensemble modelling improves model robustness by integrating the predictive strengths of different algorithms [68,69,73].

After host plant availability was integrated, the number of used individual algorithms changed to varying degrees among the three papilionids. For *L. chinensis*, *T. imperialis*, and *T. aeacus*, eight, seven, and six individual algorithms were retained, respectively (Table 1 and Table 2), indicating that the inclusion of host plant availability altered the ensemble algorithm and model applicability. Similarly, the Env+Host ensemble models were superior to most individual models. Despite achieving high predictive performance, the two sets of ensemble models showed only minor differences in performance. The AUC values of *L. chinensis* and *T. aeacus* increased slightly and their TSS decreased trivially when host plant availability was incorporated. In contrast, both AUC and TSS of *T. imperialis* decreased modestly. In addition, the slight reduction or inconsistency in model performance metrics, particularly TSS (Table 2), does not necessarily indicate that the Env+Host models are ecologically less reliable. Conventional SDM evaluation metrics such as AUC and TSS primarily assess statistical discrimination between occurrence records and background or pseudo-absence points, rather than directly evaluating whether the predicted suitable habitats are biologically accessible to the species. In this study, host plant availability is incorporated as an ecological constraint, restricting butterfly suitable habitats to areas that are both environmentally suitable and potentially capable of supporting larval feeding and development. Although this ecological filtering may slightly reduce statistical discrimination against pseudo-absence data, it can improve the biological realism and conservation applicability of the model predictions. Furthermore, the MESS-based extrapolation assessment further supported the transferability of most future projections (Table S7), because extrapolation-risk cells accounted for only a small proportion of the projected areas in most scenarios, indicating that the differences between Env-only and Env+Host projections are unlikely to be mainly driven by broad environmental extrapolation artifacts. Therefore, both modelling frameworks are employed to predict the current and future suitable habitats of the three papilionid butterflies.

In addition to model performance, host plant availability had a pronounced effect on the variable contribution patterns of the three papilionid butterflies. In the Env-only models, the leading predictors were precipitation-related variables with the highest contribution for each species. Specifically, these variables were Bio17 for *L. chinensis*, Bio18 for *T. imperialis*, and Bio12 for *T. aeacus*. This indicates that precipitation plays an important role in constraining the potential distributions of these endangered butterflies. In the Env+Host models, host plant availability shows the highest contribution for both *L. chinensis* and *T. aeacus*, with contribution rates of 41.54% and 51.53%, respectively; meanwhile, the most important factor (i.e., Bio17 and Bio12) for the two butterflies in the Env-only models ranks second correspondingly. By contrast, for *T. imperialis*, Bio18 still ranks first in both the Env+Host and the Env-only models. However, host plant availability ranks fourth, with a contribution rate of 11.31% (Table 3). These results indicate that the role of host plant availability in shaping potential distributions differs among papilionid species. For *L. chinensis* and *T. aeacus*, host-plant resources may represent important biotic constraints affecting their potential distributions. For *T. imperialis*, precipitation during the warmest quarter may serve as the dominant environmental constraint, while host plant availability may further filter its potential distribution within climatically suitable areas [17].

Such interspecific differences may be related to the extent of host-plant specialization, the distribution range of host plants, and the spatial patterns of the butterflies themselves. For monophagous or oligophagous butterflies, larval host plants not only influence where adult females choose to lay eggs but also affect whether larvae can consume successfully and complete development. Therefore, host-plant resources often contribute to limiting the realized distribution boundaries of these species [19]. In the Env+Host models for *L. chinensis* and *T. aeacus*, the higher contributions of host-plant variables suggest that their potential distributions are unlikely to be constrained by climatic conditions alone; instead, they are closely associated with the spatial availability of host plants. In the model for *T. imperialis*, Bio18 remained the top-ranked factor, consistent with the Env-only model, suggesting that its potential distribution may be primarily constrained by precipitation during the warmest quarter in the mountainous regions of southwestern China. Although it ranks only fourth in contribution for this species, host plant availability may still further restrict its local suitable areas within the broader climatically suitable region. Overall, host plant availability does not exert a similar effect on these papilionid distributions. Indeed, we argue that this factor may well interact with climatic conditions and species-specific ecological requirements in governing their suitability. Nevertheless, because all future projections in this study were derived from a single GCM, BCC-CSM2-MR, the magnitude and spatial pattern of the projected habitat changes may partly reflect model-specific climate responses. Although the MESS analysis identifies areas of environmental extrapolation, it does not quantify uncertainty of GCMs; future studies should therefore incorporate multiple GCMs or multi-model climate ensembles to test the robustness of suitable-habitat projections for both the butterflies and their host plants.

### 4.2. Changes in Suitable Ranges of Three Endangered Papilionid Butterflies

When host plant availability was incorporated, the predicted suitable areas of the three papilionid butterflies generally contracted, indicating that Env-only models may identify some climatically suitable areas without sufficient host resources as potential suitable habitats. This pattern may be partly explained by the limited mobility of butterfly larvae. Although adult butterflies can disperse among habitat patches, eggs are immobile and larvae generally have much lower movement capacity than adults; therefore, larval survival depends strongly on whether suitable host plants are available at or near oviposition sites [74,75,76]. In this context, host plant availability does not simply represent a food resource, but also determines whether climatically suitable habitats are biologically accessible to immature stages. Therefore, areas predicted as suitable by the Env-only models may become unavailable in the Env+Host models when host plants are absent or spatially discontinuous. At present, the proportions of host-related contraction areas for *L. chinensis*, *T. imperialis*, and *T. aeacus* were 32.67%, 31.12%, and 4.63%, respectively, and they were higher than the corresponding proportions of host-related expansion areas. This trend was projected to persist under future climate scenarios. The mean proportion of host-related contraction areas was 32.99% for *L. chinensis* and reached 49.41% for *T. imperialis*, whereas it was only 5.94% for *T. aeacus* (Table 4). These results indicate that incorporating host plant availability generally reduces the predicted suitable areas, thereby shifting model outputs from environmentally suitable areas toward potentially usable habitats constrained by host resources [16,17,19].

Our analysis suggests that SDMs based solely on abiotic variables, such as climate, topography, and human disturbance, may be insufficient to determine the potentially usable habitats for host-specialized papilionid butterflies. Traditional SDMs are useful for delineating broad areas of environmental suitability. However, if plant and animal interactions are ignored, model projections may overestimate the actual distribution ranges of butterfly species [24,77], especially for specialist butterflies. For papilionid butterflies, larvae are often strongly dependent on obligate host plants [19,78]. For instance, the caterpillars of *L. chinensis* exclusively feed on *Asarum forbesii* and *A. sieboldii* [35]. As a result, if its larval host plants are absent within the suitable range for a certain papilionid, this may prevent the species from establishing or maintaining stable populations owing to host specificity. Furthermore, to what extent host plant availability modifies predicted suitable areas largely depends on the spatial overlap between host plant availability and climatic suitability of papilionid butterflies.

Our analysis also indicates that the three papilionids have distinct responses of suitability after incorporating host plants into SDMs. For example, *L. chinensis* and *T. imperialis* are expected to shrink markedly in the Env+Host model under both current and future climate scenarios, with a decrease of nearly one-third (Table 4). In contrast, *T. aeacus* was observed to present great overlap between spatial coverage of suitable host plants and its climatically suitable habitat. In other words, *T. aeacus* will contract slightly in the same conditions, with a reduction of approximately 5%. As a whole, this could be ascribed to distribution differences between butterflies and host plants, as well as host plant resources (i.e., species, abundance, and accessibility) [20,79].

### 4.3. Host-Mediated Changes in Habitat Quality

This study further shows that host plant availability not only reduces the potential suitable habitats of butterflies, but also reshapes the spatial configuration and structural characteristics of the suitable habitats, as suggested by the landscape pattern metrics used in this study. For *L. chinensis*, except for the number of patches (i.e., NP) under the current condition, the other landscape metrics (i.e., MPA, LPI, and SPLIT) under both current and future scenarios congruently showcased more fragmented habitats in the Env+Host models than in Env-only models (Table 5). The decline in NP may indicate that some small and isolated peripheral patches entirely disappeared after host plant availability was incorporated. However, this does not necessarily imply an improvement in habitat continuity. Instead, the simultaneous increase in SPLIT, together with the marked decreases in MPA and LPI, suggests that the host-plant filter likely further divides some formerly large and continuous suitable habitats into smaller and more spatially separated patches. Therefore, these combined changes indicate an overall increase in habitat fragmentation. For *T. imperialis*, incorporating host plant availability also resulted in greater fragmentation of suitable habitats under both scenarios. In contrast, *T. aeacus* showed enhanced spatial continuity among the retained suitable habitats in the Env+Host models. Overall, after accounting for host plant availability, *L. chinensis* and *T. imperialis* became more fragmented whereas *T. aeacus* became more spatially concentrated in suitable habitats. Therefore, these papilionids likely undergo divergent changes in the spatial structure of suitable habitats when constrained by host plant availability.

This divergence may be mainly related to interspecific differences in host-plant dependence, dispersal ability, and known geographic distributions among the three papilionid butterflies. As a univoltine butterfly, *L. chinensis* has a short adult stage of 2–4 weeks, with a poor dispersal capability. Moreover, its larvae have strong reliance on host plants, exclusively consuming two *Asarum* species. These perennial herbs grow well in the shady and moist understory of subtropical forests in China. Therefore, host plants are patchily distributed within climatically suitable areas for this butterfly in the Env-only models, and these continuous habitats are very likely to be split into more isolated or smaller patches, thereby resulting in increased habitat fragmentation [80]. *T. imperialis* usually inhabits middle and high elevation montane forests (i.e., 1500–3000 m) [1]. Because this papilionid depends on well-preserved mountain forest habitats and specific host plant resources [81], the spatial discontinuity of these habitats likely restricts the available habitat range for the butterfly. As a result, its suitable area becomes even more fragmented when host plants are taken into account. Compared with the two butterflies, *T. aeacus* is more widely distributed, with most records from southern China, northern India, Nepal and some other countries of Southeast Asia [40,82]. Its larvae are able to feed on multiple *Aristolochia* species, suggesting that its host-plant resources are more abundant. Therefore, after host plants are incorporated into the models, the excluded areas may mainly represent marginal patches without host resources while the core suitable patches may be retained within such areas with host resources. In addition, our results can be interpreted in accordance with the stress-gradient hypothesis, which contends that abiotic factors tend to shape species distribution limits under physiologically stressful environmental conditions, whereas negative biotic interactions may become more important under less stressful conditions [19]. In general, host plants are key resources for feeding and oviposition of papilionids. Compared with *L. chinensis* and *T. imperialis*, *T. aeacus* has higher larval host-plant richness (Table S2), together with larger distribution range (Figure 4); therefore, it displays relatively continuous suitable habitat.

Combining landscape pattern metrics with host-plant availability in SDMs, it is effective to reflect spatial differences among suitable habitats in terms of patch number and size, spatial aggregation, and landscape splitting, thereby contributing to revealing potential habitat quality for butterflies.

### 4.4. Conservation Implications for Endangered Papilionid Butterflies in China

Our analysis demonstrates that larval host-plant availability should be incorporated into SDMs to assess the potential suitable habitats for endangered papilionid butterflies, rather than solely by Env-only models. The great majority of papilionid butterflies depend strongly on larval host plants [83,84]. Such hosts provide the nutritional components for larval feeding, growth, and development, and they produce specific chemical compounds that can serve as important cues for oviposition, larval host recognition, and defensive compound accumulation [84,85]. For example, aristolochic acids and other secondary metabolites may function as oviposition stimulants or chemical defense for swallowtail butterflies which feed on Aristolochiaceae plants [86,87]. Therefore, the absence of key host plants may compromise population establishment and long-term persistence even if there are climatically suitable habitats for butterflies. In addition, the distribution range of target species should be accounted for when incorporating host plant availability into the Env+Host model. SDMs can generally identify climatically suitable areas for species at the macro-scale. However, when the geographic range of a target species is relatively small (i.e., <10 km^2^), biotic interactions become one of the important factors limiting its geographic distribution [17]. Such an effect is also related to the species-specific ecological traits [16]. In the Env-only models, precipitation-related variables were among the dominant predictors for all three endangered papilionid butterflies. After host plant availability was incorporated, precipitation of the warmest quarter remained the most important predictor for *T. imperialis*. These results suggest that suitable moisture conditions may be a key consideration when improving habitat quality within potentially suitable areas. Adequate moisture can help maintain local vegetation and the availability of larval host plants, while also creating favorable microhabitat conditions for butterfly oviposition, larval feeding, growth, and development. Based on our model projections and habitat-overlap analyses, we propose to adopt distinct conservation and management strategies in response to suitable habitat shifts of these endangered papilionids under climate change. On the one hand, given that these focal butterflies are listed as nationally protected wildlife species in China [29], we recommend setting the stable suitable areas jointly identified by the Env-only and Env+Host models as conservation priorities and conducting long-term monitoring [2]. Specifically, as for the stable suitable areas *L. chinensis* is mainly located in the mountainous regions of central-eastern to southeastern China, *T. imperialis* is primarily concentrated in the mountains of southwestern China (Figure 3), and *T. aeacus* is mainly distributed in southern China, forming a relatively continuous core suitable area (Figure 4). On the other hand, as for host-related contraction areas identified by the overlay analysis, we recommend protecting or restoring suitable microhabitats, re-establishing locally larval host plants [88]. These areas represent habitats that may remain climatically suitable for the butterflies but biologically inaccessible because of insufficient host resources (Table 4; Figure 2, Figure 3 and Figure 4). In addition to climate change and host plant availability, anthropogenic land-use change may also give rise to population decline and habitat loss of these threatened papilionid butterflies. The conversion of natural habitats into agricultural land, urban expansion, road construction, and other infrastructure development may directly reduce and fragment their suitable habitats. Also, they may degrade native vegetation or even cause the local disappearance of larval host plants. For host-specialized butterflies, the host plant loss probably renders climatically suitable areas unavailable for oviposition, larval feeding, and long-term population persistence.

Except for their suitable habitats, it should be taken into account their spatial connectivity of patches when protecting these threatened papilionids. Increased habitat fragmentation can reduce the size of local habitat patches, increase isolation among patches, and limit individual movement among suitable areas. For endangered butterfly species that persist as spatially structured populations, such reduced connectivity may weaken dispersal and recolonization among local populations, thereby increasing the risk of local extinction and reducing long-term metapopulation persistence [89,90,91]. Previous studies have shown that establishing ecological corridors and maintaining connectivity among these patches can facilitate gene flow, individual dispersal, and local population replenishment [91,92]. For *L. chinensis* and *T. imperialis*, conservation countermeasures should prioritize maintaining the integrity of community habitats, enhancing the connectivity among suitable patches, and minimizing anthropogenic disturbance within their core distribution areas [93], thereby improving the accessibility of adult butterflies. *T. aeacus* shows a high proportion of stable suitable habitat and a relatively continuous core suitable area after host plant incorporation (Table 4; Figure 4); therefore, more attention should be paid to the quality of its potential suitable habitats in southern China, particularly the availability and abundance of host plants therein.

Furthermore, because the papilionid species respond differently to climate change in suitable habitat and habitat quality, conservation efforts should be tailored to host specificity, geographic distribution, and habitat requirements of threatened papilionids in China.

## 5. Conclusions

This study demonstrates that incorporating larval host plant availability into species distribution models can substantially affect the resulting projections for the threatened papilionid butterflies. In the environment-only models, precipitation-related variables are identified as the primary factors constraining the potential distributions of all three species. After it is incorporated, host plant availability becomes the most important predictor for both *Luehdorfia chinensis* and *Troides aeacus*, whereas for *Teinopalpus imperialis*, precipitation of the warmest quarter remains the key limiting factor. Compared with the environment-only models, the host-inclusive models forecast smaller suitable habitat for each papilionid under both current and future climate scenarios and furthermore alter the spatial structure of these habitats to varying degrees. Overall, the three papilionids exhibit divergent responses after incorporation of host plant availability. Therefore, conservation planning should consider potential suitable habitats together with their spatial connectivity; meanwhile conservation efforts should be tailored to host specificity, climate-driven distribution, as well as habitat requirements of threatened papilionid butterflies in China.

## Figures and Tables

**Table 1:** AUC values of single algorithms and ensemble models with and without host plant availability.

Model	*Luehdorfia chinensis*		*Teinopalpus imperialis*		*Troides aeacus*	
	Env-only	Env+Host	Env-only	Env+Host	Env-only	Env+Host
ANN	0.505 ± 0.010	0.945 ± 0.027	0.823 ± 0.062	0.922 ± 0.137	0.000 ± 0.018	0.926 ± 0.026
CTA	0.842 ± 0.022	0.933 ± 0.048	0.760 ± 0.254	0.853 ± 0.461	0.937 ± 0.028	0.837 ± 0.034
FDA	0.928 ± 0.012	0.959 ± 0.062	0.871 ± 0.038	0.924 ± 0.126	0.921 ± 0.048	0.945 ± 0.028
GAM	0.948 ± 0.007	0.913 ± 0.040	0.954 ± 0.170	0.913 ± 0.330	0.929 ± 0.011	0.954 ± 0.021
GBM	0.913 ± 0.005	0.983 ± 0.035	0.839 ± 0.061	0.899 ± 0.106	0.957 ± 0.010	0.951 ± 0.024
GLM	0.937 ± 0.012	0.939 ± 0.049	0.664 ± 0.057	0.707 ± 0.124	0.940 ± 0.011	0.937 ± 0.038
MARS	0.941 ± 0.010	0.978 ± 0.033	0.818 ± 0.134	0.834 ± 0.171	0.952 ± 0.011	0.946 ± 0.024
MAXENT	0.822 ± 0.007	0.818 ± 0.039	0.901 ± 0.030	0.813 ± 0.083	0.936 ± 0.015	0.897 ± 0.027
RF	0.944 ± 0.005	0.987 ± 0.028	0.870 ± 0.063	0.945 ± 0.099	0.993 ± 0.011	0.957 ± 0.025
SRE	0.500 ± 0.013	0.831 ± 0.036	0.500 ± 0.019	0.559 ± 0.086	0.500 ± 0.006	0.709 ± 0.044
Ensemble model	0.980 ± 0.007	0.987 ± 0.033	0.993 ± 0.075	0.952 ± 0.146	0.904 ± 0.012	0.949 ± 0.020

**Note:** Values are presented as mean ± standard deviation across repeated model runs.

**Table 2:** TSS values of single algorithms and ensemble models with and without host plant availability.

Model	*Luehdorfia chinensis*		*Teinopalpus imperialis*		*Troides aeacus*	
	Env-only	Env+Host	Env-only	Env+Host	Env-only	Env+Host
ANN	0.561 ± 0.022	0.811 ± 0.041	0.467 ± 0.046	0.368 ± 0.115	0.000 ± 0.018	0.709 ± 0.048
CTA	0.692 ± 0.040	0.851 ± 0.042	0.549 ± 0.169	0.705 ± 0.233	0.769 ± 0.033	0.674 ± 0.064
FDA	0.816 ± 0.011	0.817 ± 0.046	0.824 ± 0.063	0.878 ± 0.149	0.707 ± 0.014	0.734 ± 0.059
GAM	0.845 ± 0.005	0.829 ± 0.031	0.933 ± 0.013	0.836 ± 0.055	0.729 ± 0.013	0.779 ± 0.040
GBM	0.760 ± 0.008	0.823 ± 0.033	0.773 ± 0.038	0.848 ± 0.112	0.798 ± 0.011	0.728 ± 0.041
GLM	0.862 ± 0.009	0.863 ± 0.039	0.352 ± 0.025	0.495 ± 0.058	0.764 ± 0.013	0.722 ± 0.022
MARS	0.855 ± 0.010	0.874 ± 0.036	0.749 ± 0.059	0.759 ± 0.111	0.789 ± 0.014	0.731 ± 0.039
MAXENT	0.719 ± 0.007	0.729 ± 0.040	0.725 ± 0.016	0.713 ± 0.032	0.748 ± 0.013	0.687 ± 0.041
RF	0.884 ± 0.006	0.698 ± 0.045	0.827 ± 0.032	0.912 ± 0.086	0.945 ± 0.011	0.697 ± 0.042
SRE	0.000 ± 0.023	0.662 ± 0.033	0.000 ± 0.039	0.119 ± 0.074	0.000 ± 0.023	0.419 ± 0.015
Ensemble model	0.930 ± 0.008	0.908 ± 0.034	0.991 ± 0.028	0.883 ± 0.053	0.769 ± 0.012	0.762 ± 0.031

**Note:** Values are presented as mean ± standard deviation across repeated model runs.

**Table 3:** Top four predictors in the environment-only models and shifts in their percentage contributions after incorporating host plant availability for the three papilionid butterflies.

Species	Environmental Predictor (Without Host) (%, Contribution)	Environmental Predictor (with Host) (%, Contribution)
*Luehdorfia chinensis*	Bio17 (54.88)	Host plant availability (41.54)
	Bio04 (17.58)	Bio17 (19.47)
	HI (10.01)	Bio04 (18.09)
	Bio09 (6.94)	HI (7.73)
*Teinopalpus imperialis*	Bio18 (34.51)	Bio18 (36.00)
	Bio07 (20.00)	Bio07 (16.93)
	Slope (12.53)	Slope (12.98)
	Bio10 (8.15)	Host plant availability (11.31)
*Troides aeacus*	Bio12 (52.37)	Host plant availability (51.53)
	Bio07 (19.75)	Bio07 (20.34)
	Bio06 (13.88)	Bio12 (15.72)
	HI (4.93)	HI (4.12)

**Table 4:** Comparison of the proportions of stable, expanded, and contracted suitable habitats of three papilionid butterflies between models with and without host plant availability under different climate scenarios.

Climate Scenario	*Luehdorfia chinensis*			*Teinopalpus imperialis*			*Troides aeacus*		
	Stable Suitable (%)	Expansion (%)	Contraction (%)	Stable Suitable (%)	Expansion (%)	Contraction (%)	Stable Suitable (%)	Expansion (%)	Contraction (%)
Current	65.64	1.70	32.67	49.30	19.58	31.12	93.93	1.44	4.63
2050–SSP126	59.97	5.18	34.85	31.89	1.45	66.67	94.58	0.99	4.43
2050–SSP245	55.12	3.02	41.86	51.96	41.85	6.18	93.94	1.13	4.94
2050–SSP585	63.25	5.47	31.27	37.69	1.73	60.58	93.87	1.31	4.82
2070–SSP126	58.59	6.42	34.99	45.85	18.86	35.29	92.01	1.82	6.18
2070–SSP245	62.76	5.77	31.47	53.82	29.81	16.37	93.67	1.92	4.41
2070–SSP585	63.97	6.02	30.01	32.73	2.79	64.48	89.24	2.28	8.47
2090–SSP126	60.90	8.01	31.09	35.41	2.49	62.10	91.16	1.47	7.37
2090–SSP245	62.85	1.46	35.69	32.02	3.41	64.57	91.12	1.80	7.07
2090–SSP585	63.99	10.32	25.69	28.29	3.26	68.44	92.18	2.01	5.81
Mean	61.27	5.74	32.99	38.85	11.74	49.41	92.42	1.64	5.94

**Note:** The “Mean” row represents the average values across the nine future climate scenarios.

**Table 5:** Summary of current and future mean changes in landscape pattern indices of three papilionid butterflies after incorporating host availability.

Metrics	Model	*Luehdorfia chinensis*		*Teinopalpus imperialis*		*Troides aeacus*	
		Current	Future Mean	Current	Future Mean	Current	Future Mean
NP	Env-only	9931	5410.67	70	1305.11	5088	7001.89
	Env+Host	5642	6334.89	11,631	3689.89	3858	2043.56
	ΔNP (%)	−43.19	17.08	16,515.71	182.73	−24.17	−70.81
MPA (km^2^)	Env-only	8.15	16.99	1356.11	241.22	15.86	12.34
	Env+Host	2.44	2.43	0.71	2.89	6.79	13.80
	ΔMPA (%)	−70.18	−85.7	−99.95	−98.8	−57.19	11.75
LPI (%)	Env-only	24.42	24.87	29.01	27.9	23.5	24.86
	Env+Host	12.71	13.38	5.26	4.49	25.29	26.56
	ΔLPI (%)	−47.95	−46.16	−81.87	−83.91	7.62	6.84
SPLIT	Env-only	16.77	16.22	11.88	12.89	18.09	16.18
	Env+Host	61.86	56.54	353.38	1710.19	15.61	14.18
	ΔSPLIT (%)	268.87	248.58	2874.58	13,167.57	−13.71	−12.36

**Note:** NP = number of patches; MPA = mean patch area; LPI = largest patch index; SPLIT = splitting index. “Env-only” refers to models constructed using only environmental variables, whereas “Env+Host” refers to models incorporating both environmental variables and host plant availability. ΔNP, ΔMPA, ΔLPI and ΔSPLIT indicate the percentage changes in the corresponding landscape pattern indices from Env-only to Env+Host, respectively. Future mean indicates the average across future climate scenarios.

## Data Availability

Data are contained within the article and Supplementary Materials.

## Supplementary Materials

The following supporting information can be downloaded at: https://www.mdpi.com/article/10.3390/biology15151232/s1, Table S1: Species distribution occurrences of the three focal papilionid butterflies and their corresponding host plants in China; Table S2: Larval host plants of the three focal papilionid butterflies in China and their use in host-availability modelling; Table S3: Occurrence records of *Luehdorfia chinensis*, *Teinopalpus imperialis*, and *Troides aeacus* in China; Table S4: Key environmental variables associated with habitat suitability of larval host plants for the three papilionid butterflies in China; Table S5: Model performances of larval host plants of the three papilionid butterflies in China; Table S6: Changes in landscape pattern indices of three papilionid butterflies in China after incorporating host availability under different climate scenarios; Table S7: MESS-based extrapolation-risk cell percentages (%) for future suitable habitat projections of three papilionid butterflies in China. Figure S1: Pearson correlation matrix among candidate environmental variables prior to collinearity screening and model construction.
