# Supplementary material for: Integrating Host Plant Availability into Ensemble Models Reveals Divergent Responses of Three Endangered Papilionid Butterflies to Climate Change in China
Source: Biology (Basel). 2026 Jul 24;15(15):1232. doi: 10.3390/biology15151232 (PMC13465108; doi:10.3390/biology15151232)
Supplement: Supplementary file 1 [file biology-15-01232-s001.zip › Table S2. Larval host plants of the three focal papilionid butterflies in China and their use in host-availability modelling..pdf]

Table S2. Larval host plants of the three focal papilionid butterflies in China and their use in host-availability modelling.

Butterfly species	Family name of host plants	Genus name of host plants	Reported larval host plant species	Availability of inclusive-host modelling
Luehdorfia chinensis	Aristolochiaceae	*Asarum*	*Asarum sieboldii*	✓
Luehdorfia chinensis	Aristolochiaceae	*Asarum*	*Asarum forbesii*	✓
Teinopalpus imperialis	Thymelaeaceae	*Daphne*	*Daphne bholua*	✓
Teinopalpus imperialis	Magnoliaceae	*Yulania*	*Yulania campbellii*	✓
Troides aeacus	Aristolochiaceae	*Aristolochia*	*Aristolochia debilis*	✓
Troides aeacus	Aristolochiaceae	*Aristolochia*	*Aristolochia kaempferi*	✓
Troides aeacus	Aristolochiaceae	*Aristolochia*	*Aristolochia zollingeriana*	✓
Troides aeacus	Aristolochiaceae	*Aristolochia*	*Aristolochia cucurbitifolia*	✓
Troides aeacus	Aristolochiaceae	*Aristolochia*	*Aristolochia liukiuensis*	✓
Troides aeacus	Aristolochiaceae	*Aristolochia*	*Aristolochia griffithii*	✓
Troides aeacus	Aristolochiaceae	*Aristolochia*	*Aristolochia cathcartii*	-
Troides aeacus	Aristolochiaceae	*Aristolochia*	*Aristolochia indica*	-
Troides aeacus	Aristolochiaceae	*Thottea*	*Thottea corymbosa*	-
Troides aeacus	Aristolochiaceae	*Thottea*	*Thottea tricornis*	-

Note: The tick (✓) in the last column indicates that the host plant species is used to construct host plant habitat-suitability models and host-availability layers; "-" refers to the species in the reported larval host plant checklist but not modelled because of insufficient reliable georeferenced records.
