# Supplementary material for: Integrating Host Plant Availability into Ensemble Models Reveals Divergent Responses of Three Endangered Papilionid Butterflies to Climate Change in China
Source: Biology (Basel). 2026 Jul 24;15(15):1232. doi: 10.3390/biology15151232 (PMC13465108; doi:10.3390/biology15151232)
Supplement: Supplementary file 1 [file biology-15-01232-s001.zip › Table S3. Occurrence records of Luehdorfia chinensis, Teinopalpus imperialis, and Troides aeacus in China..pdf]

Table S3. Occurrence records of *Luehdorfia chinensis*, *Teinopalpus imperialis*, and *Troides aeacus* in China.

Species	Longitude (°)	Latitude (°)	Species	Longitude (°)	Latitude (°)
Luehdorfia_chinensis	119.00	32.00	*Troides_aeacus*	120.70	23.24
Luehdorfia_chinensis	114.30	30.60	*Troides_aeacus*	114.07	23.26
Luehdorfia_chinensis	118.60	32.10	*Troides_aeacus*	120.73	23.33
Luehdorfia_chinensis	118.90	31.90	*Troides_aeacus*	120.63	23.38
Luehdorfia_chinensis	119.10	32.00	*Troides_aeacus*	120.66	23.42
Luehdorfia_chinensis	118.20	31.10	*Troides_aeacus*	121.37	23.45
Luehdorfia_chinensis	118.40	32.10	*Troides_aeacus*	121.26	23.48
Luehdorfia_chinensis	119.10	32.10	*Troides_aeacus*	120.43	23.49
Luehdorfia_chinensis	119.00	32.20	*Troides_aeacus*	120.63	23.61
Luehdorfia_chinensis	114.30	30.50	*Troides_aeacus*	113.96	23.79
Luehdorfia_chinensis	118.80	32.10	*Troides_aeacus*	120.70	23.80
Luehdorfia_chinensis	119.00	32.10	*Troides_aeacus*	120.87	23.88
Luehdorfia_chinensis	114.20	30.60	*Troides_aeacus*	120.92	23.89
Luehdorfia_chinensis	119.20	32.10	*Troides_aeacus*	120.91	23.96
Luehdorfia_chinensis	119.20	32.00	*Troides_aeacus*	121.59	23.99
Luehdorfia_chinensis	119.10	32.20	*Troides_aeacus*	120.79	24.01
Luehdorfia_chinensis	118.80	28.40	*Troides_aeacus*	120.66	24.04
Luehdorfia_chinensis	119.20	32.20	*Troides_aeacus*	120.69	24.07
Luehdorfia_chinensis	114.50	30.40	*Troides_aeacus*	120.71	24.08

Luehdorfia_chinensis	118.50	32.10	*Troides_aeacus*	120.79	24.09
Luehdorfia_chinensis	110.10	34.50	*Troides_aeacus*	121.03	24.11
Luehdorfia_chinensis	119.40	32.20	*Troides_aeacus*	121.23	24.16
Luehdorfia_chinensis	118.90	32.20	*Troides_aeacus*	120.76	24.58
Luehdorfia_chinensis	118.60	32.00	*Troides_aeacus*	120.90	24.61
Luehdorfia_chinensis	118.90	32.00	*Troides_aeacus*	97.66	24.61
Luehdorfia_chinensis	118.50	32.00	*Troides_aeacus*	120.93	24.72
Luehdorfia_chinensis	114.40	30.50	*Troides_aeacus*	120.95	24.73
Luehdorfia_chinensis	114.60	30.40	*Troides_aeacus*	120.93	24.75
Luehdorfia_chinensis	119.30	31.80	*Troides_aeacus*	121.60	24.80
Luehdorfia_chinensis	114.60	30.50	*Troides_aeacus*	121.48	24.80
Luehdorfia_chinensis	119.30	31.70	*Troides_aeacus*	121.44	24.80
Luehdorfia_chinensis	120.00	31.80	*Troides_aeacus*	121.42	24.81
Luehdorfia_chinensis	116.60	29.80	*Troides_aeacus*	121.48	24.81
Luehdorfia_chinensis	119.00	31.30	*Troides_aeacus*	121.54	24.83
Luehdorfia_chinensis	120.20	30.30	*Troides_aeacus*	121.43	24.84
Luehdorfia_chinensis	118.51	32.05	*Troides_aeacus*	121.56	24.84
Luehdorfia_chinensis	118.54	32.07	*Troides_aeacus*	121.47	24.85
Luehdorfia_chinensis	118.57	32.07	*Troides_aeacus*	121.58	24.89
Luehdorfia_chinensis	118.62	32.11	*Troides_aeacus*	121.59	24.91
Luehdorfia_chinensis	118.79	32.02	*Troides_aeacus*	121.40	24.92
Luehdorfia_chinensis	118.85	28.40	*Troides_aeacus*	121.51	24.92

Luehdorfia_chinensis	118.96	32.00	*Troides_aeacus*	121.45	24.93
Luehdorfia_chinensis	119.03	32.06	*Troides_aeacus*	121.48	24.96
Luehdorfia_chinensis	119.11	32.12	*Troides_aeacus*	121.49	24.97
Luehdorfia_chinensis	119.12	32.18	*Troides_aeacus*	121.58	24.97
Luehdorfia_chinensis	119.19	32.12	*Troides_aeacus*	121.47	24.99
Luehdorfia_chinensis	108.78	33.85	*Troides_aeacus*	102.83	25.08
Luehdorfia_chinensis	107.18	34.03	*Troides_aeacus*	100.15	25.67
Luehdorfia_chinensis	108.18	33.19	*Troides_aeacus*	100.13	25.69
Luehdorfia_chinensis	115.00	31.15	*Troides_aeacus*	100.14	25.69
Luehdorfia_chinensis	110.40	31.44	*Troides_aeacus*	113.12	25.76
Luehdorfia_chinensis	115.52	29.26	*Troides_aeacus*	118.08	26.84
Luehdorfia_chinensis	111.12	28.32	*Troides_aeacus*	92.98	26.94
Luehdorfia_chinensis	111.16	28.31	*Troides_aeacus*	92.47	27.21
Luehdorfia_chinensis	111.18	28.31	*Troides_aeacus*	93.82	27.34
Luehdorfia_chinensis	111.21	28.32	*Troides_aeacus*	92.30	27.40
Luehdorfia_chinensis	111.22	28.33	*Troides_aeacus*	117.78	27.62
Luehdorfia_chinensis	111.25	28.33	*Troides_aeacus*	117.66	27.71
Luehdorfia_chinensis	108.19	33.84	*Troides_aeacus*	98.33	27.86
Luehdorfia_chinensis	107.90	34.15	*Troides_aeacus*	117.88	27.87
Luehdorfia_chinensis	108.48	33.78	*Troides_aeacus*	95.72	28.27
Luehdorfia_chinensis	110.08	34.53	*Troides_aeacus*	107.15	28.28
Luehdorfia_chinensis	115.67	31.09	*Troides_aeacus*	94.63	28.38

Luehdorfia_chinensis	121.03	28.38	*Troides_aeacus*	103.25	28.57
Luehdorfia_chinensis	119.42	30.35	*Troides_aeacus*	95.04	28.62
Luehdorfia_chinensis	111.35	28.54	*Troides_aeacus*	94.98	28.86
Luehdorfia_chinensis	111.37	28.56	*Troides_aeacus*	115.09	28.88
Luehdorfia_chinensis	111.38	28.56	*Troides_aeacus*	117.62	29.48
Luehdorfia_chinensis	119.02	32.07	*Troides_aeacus*	102.88	29.63
Luehdorfia_chinensis	118.83	32.07	*Troides_aeacus*	102.20	29.68
Luehdorfia_chinensis	119.01	32.07	*Troides_aeacus*	119.09	29.76
Luehdorfia_chinensis	119.02	32.06	*Troides_aeacus*	102.85	29.79
Luehdorfia_chinensis	118.55	32.05	*Troides_aeacus*	102.54	30.13
Luehdorfia_chinensis	119.05	32.05	*Troides_aeacus*	102.50	30.28
Luehdorfia_chinensis	119.08	32.13	*Troides_aeacus*	111.06	30.81
Luehdorfia_chinensis	119.02	32.05	*Troides_aeacus*	111.50	30.94
Luehdorfia_chinensis	119.18	32.02	*Troides_aeacus*	111.51	30.98
Luehdorfia_chinensis	118.88	31.89	*Troides_aeacus*	116.08	31.01
Luehdorfia_chinensis	119.06	32.07	*Troides_aeacus*	110.93	31.06
Luehdorfia_chinensis	119.10	32.15	*Troides_aeacus*	119.49	31.19
Luehdorfia_chinensis	119.18	32.05	*Troides_aeacus*	110.39	31.47
Luehdorfia_chinensis	114.20	30.56	*Troides_aeacus*	108.41	31.49
Luehdorfia_chinensis	119.09	32.05	*Troides_aeacus*	108.57	31.59
Luehdorfia_chinensis	118.80	32.06	*Troides_aeacus*	110.89	31.66
Luehdorfia_chinensis	119.06	32.06	*Troides_aeacus*	114.39	31.84

Luehdorfia_chinensis	114.30	30.54	*Troides_aeacus*	105.00	32.52
Luehdorfia_chinensis	119.04	32.16	*Troides_aeacus*	112.53	32.99
Luehdorfia_chinensis	119.03	32.18	*Troides_aeacus*	105.59	33.33
Luehdorfia_chinensis	119.12	32.08	*Troides_aeacus*	105.60	33.33
Luehdorfia_chinensis	118.42	32.12	*Troides_aeacus*	107.83	33.43
Luehdorfia_chinensis	118.24	31.07	*Troides_aeacus*	111.94	33.51
Luehdorfia_chinensis	119.09	32.02	*Troides_aeacus*	107.98	33.51
Luehdorfia_chinensis	119.03	32.01	*Troides_aeacus*	107.82	33.55
Luehdorfia_chinensis	118.94	32.01	*Troides_aeacus*	107.54	33.59
Luehdorfia_chinensis	120.20	30.25	*Troides_aeacus*	107.77	33.59
Luehdorfia_chinensis	118.79	32.07	*Troides_aeacus*	106.89	33.64
Luehdorfia_chinensis	119.43	32.16	*Troides_aeacus*	106.79	33.66
Luehdorfia_chinensis	119.02	32.18	*Troides_aeacus*	111.62	33.67
Luehdorfia_chinensis	119.12	32.11	*Troides_aeacus*	107.97	33.76
Luehdorfia_chinensis	119.18	32.18	*Troides_aeacus*	111.59	33.85
Luehdorfia_chinensis	119.17	32.08	*Troides_aeacus*	106.97	34.23
Luehdorfia_chinensis	119.03	32.16	*Troides_aeacus*	105.21	32.67
Luehdorfia_chinensis	119.09	32.17	*Troides_aeacus*	105.87	34.56
Luehdorfia_chinensis	114.54	30.40	*Troides_aeacus*	104.59	32.90
Luehdorfia_chinensis	119.20	32.18	*Troides_aeacus*	98.35	27.74
Luehdorfia_chinensis	119.01	32.11	*Troides_aeacus*	117.99	27.69
Luehdorfia_chinensis	119.11	32.02	*Troides_aeacus*	98.62	28.02

Luehdorfia_chinensis	119.08	32.04	*Troides_aeacus*	100.80	22.01
Luehdorfia_chinensis	119.03	32.15	*Troides_aeacus*	121.20	23.00
Luehdorfia_chinensis	119.08	32.08	*Troides_aeacus*	108.30	32.90
Luehdorfia_chinensis	119.18	32.06	*Troides_aeacus*	120.10	30.30
Luehdorfia_chinensis	119.02	32.08	*Troides_aeacus*	114.10	22.30
Luehdorfia_chinensis	119.14	32.08	*Troides_aeacus*	114.10	22.20
Luehdorfia_chinensis	118.58	32.01	*Troides_aeacus*	113.90	22.20
Luehdorfia_chinensis	118.55	32.08	*Troides_aeacus*	114.20	22.20
Luehdorfia_chinensis	119.10	32.13	*Troides_aeacus*	114.10	22.40
Luehdorfia_chinensis	116.63	29.82	*Troides_aeacus*	120.70	21.80
Luehdorfia_chinensis	119.15	32.15	*Troides_aeacus*	113.90	22.30
Luehdorfia_chinensis	118.59	32.11	*Troides_aeacus*	114.20	22.30
Luehdorfia_chinensis	118.93	32.18	*Troides_aeacus*	121.00	21.90
Luehdorfia_chinensis	118.84	32.07	*Troides_aeacus*	114.40	31.80
Luehdorfia_chinensis	119.99	31.81	*Troides_aeacus*	114.20	22.50
Luehdorfia_chinensis	119.31	31.78	*Troides_aeacus*	114.20	22.40
Luehdorfia_chinensis	118.86	31.90	*Troides_aeacus*	121.20	22.60
Luehdorfia_chinensis	114.44	30.51	*Troides_aeacus*	120.60	23.40
Luehdorfia_chinensis	114.56	30.42	*Troides_aeacus*	121.40	23.40
Luehdorfia_chinensis	118.96	31.27	*Troides_aeacus*	114.00	22.40
Luehdorfia_chinensis	119.32	31.79	*Troides_aeacus*	114.30	22.40
Luehdorfia_chinensis	118.52	32.06	*Troides_aeacus*	121.80	24.20

Luehdorfia_chinensis	119.31	31.75	*Troides_aeacus*	121.90	24.70
Luehdorfia_chinensis	119.31	31.79	*Troides_aeacus*	120.30	22.30
Luehdorfia_chinensis	114.55	30.54	*Troides_aeacus*	120.90	24.70
Luehdorfia_chinensis	118.78	32.06	*Troides_aeacus*	114.00	22.30
Luehdorfia_chinensis	118.58	32.11	*Troides_aeacus*	120.70	23.70
Luehdorfia_chinensis	118.85	31.65	*Troides_aeacus*	121.20	23.20
Luehdorfia_chinensis	118.80	31.62	*Troides_aeacus*	121.50	22.70
Luehdorfia_chinensis	110.25	31.47	*Troides_aeacus*	120.80	24.60
Luehdorfia_chinensis	115.71	31.12	*Troides_aeacus*	121.30	25.10
Luehdorfia_chinensis	115.97	29.52	*Troides_aeacus*	120.20	22.80
Luehdorfia_chinensis	119.45	30.32	*Troides_aeacus*	121.60	22.70
Luehdorfia_chinensis	120.62	27.64	*Troides_aeacus*	121.70	24.20
Luehdorfia_chinensis	118.85	32.07	*Troides_aeacus*	121.10	23.00
Luehdorfia_chinensis	118.97	31.25	*Troides_aeacus*	120.30	22.60
Luehdorfia_chinensis	118.74	31.90	*Troides_aeacus*	120.40	22.80
Luehdorfia_chinensis	118.85	32.05	*Troides_aeacus*	120.70	22.70
Luehdorfia_chinensis	120.10	30.24	*Troides_aeacus*	120.60	21.90
Luehdorfia_chinensis	116.35	29.48	*Troides_aeacus*	121.20	23.50
Luehdorfia_chinensis	119.51	30.35	*Troides_aeacus*	120.90	21.90
Luehdorfia_chinensis	119.10	32.00	*Troides_aeacus*	121.40	25.00
Luehdorfia_chinensis	114.30	30.40	*Troides_aeacus*	120.90	24.80
Teinopalpus_imperialis	98.35	27.74	*Troides_aeacus*	120.80	21.80

Teinopalpus_imperialis	103.88	31.19	*Troides_aeacus*	120.00	23.00
Teinopalpus_imperialis	102.30	29.80	*Troides_aeacus*	120.70	22.60
Teinopalpus_imperialis	101.80	29.60	*Troides_aeacus*	103.40	28.40
Teinopalpus_imperialis	102.50	31.60	*Troides_aeacus*	121.10	24.00
Teinopalpus_imperialis	103.21	22.91	*Troides_aeacus*	120.60	23.60
Teinopalpus_imperialis	103.61	28.17	*Troides_aeacus*	120.70	23.40
Teinopalpus_imperialis	101.72	29.56	*Troides_aeacus*	121.50	24.80
Teinopalpus_imperialis	103.65	30.99	*Troides_aeacus*	121.00	24.60
Teinopalpus_imperialis	103.67	30.63	*Troides_aeacus*	120.90	21.80
Teinopalpus_imperialis	103.61	31.00	*Troides_aeacus*	120.80	24.00
Teinopalpus_imperialis	103.34	29.55	*Troides_aeacus*	108.40	33.30
Teinopalpus_imperialis	102.80	24.97	*Troides_aeacus*	121.00	24.00
Teinopalpus_imperialis	103.17	30.63	*Troides_aeacus*	120.40	22.70
Teinopalpus_imperialis	101.83	29.41	*Troides_aeacus*	121.00	21.80
Teinopalpus_imperialis	102.83	30.20	*Troides_aeacus*	121.00	22.60
Teinopalpus_imperialis	106.40	24.49	*Troides_aeacus*	121.40	22.70
Teinopalpus_imperialis	101.96	30.05	*Troides_aeacus*	120.30	22.80
Teinopalpus_imperialis	98.58	25.55	*Troides_aeacus*	120.80	24.50
Teinopalpus_imperialis	107.10	29.20	*Troides_aeacus*	120.30	22.70
Troides_aeacus	113.10	24.90	*Troides_aeacus*	120.40	23.10
Troides_aeacus	101.30	21.90	*Troides_aeacus*	121.50	24.90
Troides_aeacus	117.70	27.70	*Troides_aeacus*	121.30	23.40

Troides_aeacus	113.10	25.80	*Troides_aeacus*	120.50	22.80
Troides_aeacus	107.00	34.20	*Troides_aeacus*	121.60	24.90
Troides_aeacus	95.40	29.40	*Troides_aeacus*	121.30	23.70
Troides_aeacus	107.80	33.60	*Troides_aeacus*	120.80	23.80
Troides_aeacus	108.40	31.50	*Troides_aeacus*	120.70	23.60
Troides_aeacus	100.10	25.70	*Troides_aeacus*	102.50	30.10
Troides_aeacus	107.20	28.30	*Troides_aeacus*	117.90	27.90
Troides_aeacus	109.80	18.70	*Troides_aeacus*	121.40	23.50
Troides_aeacus	108.00	33.50	*Troides_aeacus*	120.90	22.00
Troides_aeacus	115.10	28.90	*Troides_aeacus*	121.60	25.00
Troides_aeacus	97.70	24.60	*Troides_aeacus*	120.90	22.10
Troides_aeacus	108.00	21.80	*Troides_aeacus*	120.40	23.00
Troides_aeacus	108.00	33.80	*Troides_aeacus*	103.30	29.50
Troides_aeacus	105.00	32.50	*Troides_aeacus*	101.30	22.10
Troides_aeacus	116.10	31.00	*Troides_aeacus*	121.00	24.70
Troides_aeacus	100.80	22.00	*Troides_aeacus*	121.20	23.40
Troides_aeacus	98.30	27.80	*Troides_aeacus*	120.80	22.00
Troides_aeacus	114.10	23.30	*Troides_aeacus*	120.90	22.40
Troides_aeacus	118.10	26.80	*Troides_aeacus*	121.10	24.80
Troides_aeacus	117.80	27.60	*Troides_aeacus*	121.50	25.00
Troides_aeacus	117.60	29.50	*Troides_aeacus*	120.60	24.00
Troides_aeacus	102.20	29.70	*Troides_aeacus*	121.10	22.80

Troides_aeacus	103.00	29.70	*Troides_aeacus*	121.10	22.90
Troides_aeacus	119.10	29.80	*Troides_aeacus*	121.16	22.80
Troides_aeacus	118.00	28.90	*Troides_aeacus*	121.60	24.00
Troides_aeacus	115.80	31.20	*Troides_aeacus*	121.60	24.10
Troides_aeacus	98.80	25.30	*Troides_aeacus*	121.50	23.80
Troides_aeacus	109.70	30.90	*Troides_aeacus*	120.70	22.00
Troides_aeacus	111.60	33.70	*Troides_aeacus*	121.40	23.60
Troides_aeacus	111.60	33.80	*Troides_aeacus*	120.60	22.90
Troides_aeacus	116.90	26.60	*Troides_aeacus*	109.60	19.20
Troides_aeacus	109.90	18.70	*Troides_aeacus*	102.00	30.10
Troides_aeacus	114.50	27.60	*Troides_aeacus*	121.00	23.70
Troides_aeacus	111.90	33.50	*Troides_aeacus*	120.90	23.80
Troides_aeacus	107.50	33.60	*Troides_aeacus*	121.10	23.60
Troides_aeacus	110.90	31.70	*Troides_aeacus*	120.30	23.20
Troides_aeacus	110.90	31.10	*Troides_aeacus*	120.30	23.10
Troides_aeacus	108.60	31.60	*Troides_aeacus*	108.91	18.72
Troides_aeacus	98.30	27.90	*Troides_aeacus*	109.91	18.66
Troides_aeacus	110.20	20.00	*Troides_aeacus*	109.69	18.90
Troides_aeacus	111.50	30.90	*Troides_aeacus*	110.20	18.74
Troides_aeacus	110.30	31.50	*Troides_aeacus*	109.62	18.26
Troides_aeacus	111.50	31.00	*Troides_aeacus*	110.54	20.01
Troides_aeacus	110.40	31.50	*Troides_aeacus*	109.16	18.33

Troides_aeacus	111.10	30.80	*Troides_aeacus*	109.57	19.03
Troides_aeacus	114.10	22.60	*Troides_aeacus*	109.75	19.17
Troides_aeacus	119.50	31.20	*Troides_aeacus*	109.72	18.61
Troides_aeacus	102.90	29.60	*Troides_aeacus*	112.57	23.15
Troides_aeacus	119.40	31.40	*Troides_aeacus*	113.59	24.47
Troides_aeacus	110.30	20.00	*Troides_aeacus*	113.55	22.32
Troides_aeacus	102.80	25.10	*Troides_aeacus*	113.34	22.00
Troides_aeacus	109.87	18.73	*Troides_aeacus*	110.03	20.70
Troides_aeacus	108.84	18.74	*Troides_aeacus*	113.66	23.65
Troides_aeacus	110.24	20.02	*Troides_aeacus*	113.36	24.12
Troides_aeacus	110.30	20.02	*Troides_aeacus*	112.95	22.82
Troides_aeacus	101.60	21.53	*Troides_aeacus*	111.55	21.93
Troides_aeacus	108.05	21.76	*Troides_aeacus*	113.89	23.64
Troides_aeacus	101.27	21.92	*Troides_aeacus*	110.99	22.14
Troides_aeacus	101.25	21.93	*Troides_aeacus*	111.94	22.71
Troides_aeacus	101.25	21.94	*Troides_aeacus*	117.14	23.44
Troides_aeacus	120.81	21.98	*Troides_aeacus*	107.88	21.88
Troides_aeacus	100.82	22.01	*Troides_aeacus*	110.13	25.77
Troides_aeacus	120.87	22.13	*Troides_aeacus*	110.12	23.97
Troides_aeacus	113.57	22.14	*Troides_aeacus*	110.49	25.86
Troides_aeacus	114.13	22.20	*Troides_aeacus*	106.96	22.47
Troides_aeacus	120.75	22.21	*Troides_aeacus*	106.65	23.02

Troides_aeacus	113.98	22.24	*Troides_aeacus*	108.32	23.50
Troides_aeacus	114.18	22.24	*Troides_aeacus*	107.07	22.18
Troides_aeacus	113.92	22.25	*Troides_aeacus*	110.73	26.27
Troides_aeacus	114.16	22.25	*Troides_aeacus*	111.21	24.25
Troides_aeacus	113.90	22.25	*Troides_aeacus*	106.93	22.71
Troides_aeacus	114.15	22.26	*Troides_aeacus*	109.53	22.67
Troides_aeacus	113.99	22.26	*Troides_aeacus*	106.48	24.82
Troides_aeacus	113.93	22.27	*Troides_aeacus*	109.11	21.04
Troides_aeacus	114.13	22.27	*Troides_aeacus*	121.40	29.11
Troides_aeacus	113.95	22.27	*Troides_aeacus*	107.01	22.33
Troides_aeacus	114.16	22.28	*Troides_aeacus*	106.91	22.81
Troides_aeacus	114.14	22.28	*Troides_aeacus*	109.21	21.67
Troides_aeacus	113.93	22.28	*Troides_aeacus*	117.69	27.75
Troides_aeacus	113.92	22.29	*Troides_aeacus*	116.94	26.17
Troides_aeacus	113.93	22.31	*Troides_aeacus*	120.35	26.95
Troides_aeacus	114.18	22.32	*Troides_aeacus*	117.12	24.63
Troides_aeacus	114.17	22.32	*Troides_aeacus*	118.13	25.13
Troides_aeacus	114.22	22.33	*Troides_aeacus*	118.15	24.80
Troides_aeacus	114.10	22.34	*Troides_aeacus*	117.51	26.67
Troides_aeacus	114.26	22.37	*Troides_aeacus*	117.10	24.67
Troides_aeacus	114.14	22.38	*Troides_aeacus*	117.43	26.02
Troides_aeacus	114.11	22.38	*Troides_aeacus*	118.34	27.05

Troides_aeacus	114.27	22.39	*Troides_aeacus*	119.30	25.72
Troides_aeacus	114.16	22.39	*Troides_aeacus*	98.50	25.22
Troides_aeacus	114.28	22.40	*Troides_aeacus*	100.14	25.68
Troides_aeacus	114.32	22.41	*Troides_aeacus*	101.10	22.61
Troides_aeacus	114.08	22.42	*Troides_aeacus*	103.33	24.82
Troides_aeacus	114.09	22.42	*Troides_aeacus*	103.42	24.41
Troides_aeacus	114.11	22.42	*Troides_aeacus*	101.77	25.86
Troides_aeacus	114.12	22.43	*Troides_aeacus*	97.89	23.99
Troides_aeacus	114.18	22.43	*Troides_aeacus*	100.78	27.68
Troides_aeacus	114.04	22.43	*Troides_aeacus*	104.25	23.40
Troides_aeacus	114.12	22.44	*Troides_aeacus*	99.94	22.56
Troides_aeacus	114.17	22.44	*Troides_aeacus*	107.75	25.26
Troides_aeacus	114.19	22.44	*Troides_aeacus*	108.29	28.65
Troides_aeacus	114.09	22.44	*Troides_aeacus*	108.12	27.11
Troides_aeacus	114.01	22.44	*Troides_aeacus*	105.75	28.38
Troides_aeacus	114.18	22.45	*Troides_aeacus*	105.91	26.77
Troides_aeacus	114.16	22.45	*Troides_aeacus*	106.62	26.05
Troides_aeacus	114.17	22.46	*Troides_aeacus*	107.99	26.57
Troides_aeacus	114.08	22.46	*Troides_aeacus*	105.67	25.99
Troides_aeacus	114.18	22.47	*Troides_aeacus*	106.69	26.34
Troides_aeacus	120.63	22.48	*Troides_aeacus*	106.75	27.10
Troides_aeacus	114.05	22.49	*Troides_aeacus*	106.16	25.68

Troides_aeacus	114.01	22.49	*Troides_aeacus*	102.28	27.84
Troides_aeacus	114.12	22.49	*Troides_aeacus*	103.49	29.61
Troides_aeacus	114.11	22.49	*Troides_aeacus*	93.08	29.05
Troides_aeacus	114.01	22.50	*Troides_aeacus*	95.77	29.87
Troides_aeacus	114.10	22.52	*Troides_aeacus*	112.68	27.28
Troides_aeacus	114.08	22.53	*Troides_aeacus*	110.44	29.32
Troides_aeacus	114.01	22.54	*Troides_aeacus*	113.12	29.15
Troides_aeacus	114.14	22.55	*Troides_aeacus*	113.09	28.25
Troides_aeacus	114.09	22.58	*Troides_aeacus*	110.80	26.36
Troides_aeacus	114.12	22.60	*Troides_aeacus*	112.10	26.81
Troides_aeacus	120.63	22.71	*Troides_aeacus*	113.32	25.87
Troides_aeacus	120.67	22.77	*Troides_aeacus*	117.00	28.09
Troides_aeacus	120.30	22.78	*Troides_aeacus*	118.08	28.91
Troides_aeacus	120.96	22.79	*Troides_aeacus*	114.14	27.50
Troides_aeacus	120.55	22.89	*Troides_aeacus*	115.98	29.57
Troides_aeacus	120.63	22.95	*Troides_aeacus*	114.91	27.04
Troides_aeacus	121.03	23.16	*Troides_aeacus*	106.22	33.51
Troides_aeacus	121.21	23.17	*Troides_aeacus*	105.96	34.10
Troides_aeacus	121.24	23.18	*Troides_aeacus*	106.70	32.56
Troides_aeacus	113.37	23.18	*Troides_aeacus*	111.62	33.79
Troides_aeacus	120.63	23.21	*Troides_aeacus*	106.92	33.54
Troides_aeacus	113.38	23.22	*Troides_aeacus*	115.00	27.12

Troides_aeacus	120.63	23.22
