# Supplementary material for: Integrating Host Plant Availability into Ensemble Models Reveals Divergent Responses of Three Endangered Papilionid Butterflies to Climate Change in China
Source: Biology (Basel). 2026 Jul 24;15(15):1232. doi: 10.3390/biology15151232 (PMC13465108; doi:10.3390/biology15151232)
Supplement: Supplementary file 1 [file biology-15-01232-s001.zip › Table S4. Key environmental variables associated with habitat suitability of larval host plants for the three papilionid butterflies in China..pdf]

Table S4. Key environmental variables associated with habitat suitability of larval host plants for the three papilionid butterflies in China.

Butterfly species	Host plant	Environmental variables after collinearity screening	Top four environmental variables (%)
Luehdorfia chinensis	*Asarum sieboldii*	Aspect, Bio02, Bio03, Bio07, Bio09, Bio10, Bio14, Bio15, Bio18, HI, slope	Bio2 (25.21%); Bio7 (16.57%); Bio3 (10.77%); HI (9.05%)
Luehdorfia chinensis	*Asarum forbesii*	Aspect, Bio01, Bio03, Bio04, Bio14, Bio15, HI, slope	Bio14 (35.95%); Bio4 (14.80%); HI (14.09%); Bio1 (12.26%)
Teinopalpus imperialis	*Daphne bholua*	Aspect, Bio02, Bio03, Bio07, Bio09, Bio10, Bio14, Bio15, Bio18, HI, slope	Bio7 (17.30%); Bio18 (12.31%); Bio3 (11.56%); Bio2 (10.41%)
Teinopalpus imperialis	*Yulania campbellii*	Aspect, Bio02, Bio03, Bio07, Bio09, Bio10, Bio14, Bio15, Bio18, HI, slope	Bio3 (18.99%); Bio7 (17.63%); Bio2 (11.23%); Bio18 (10.41%)
Troides aeacus	*Aristolochia debilis*	Aspect, Bio02, Bio03, Bio04, Bio06, Bio14, Bio15, elevation, HI, slope	Bio6 (29.78%); Bio14 (13.83%); Bio4 (13.58%); Bio2 (13.15%)
Troides aeacus	*Aristolochia kaempferi*	Aspect, Bio03, Bio06, Bio07, Bio12, Bio15, elevation, HI, slope	Bio7 (30.21%); Bio12 (14.46%); Elevation (12.49%); Bio3 (10.67%)
Troides aeacus	*Aristolochia zollingeriana*	Aspect, Bio02, Bio03, Bio07, Bio08, Bio09, Bio15, Bio19, HI, slope	Bio7 (29.70%); Bio19 (19.74%); Bio2 (12.14%); Bio9 (9.70%)
Troides aeacus	*Aristolochia cucurbitifolia*	Aspect, Bio03, Bio06, Bio07, Bio08, Bio15, Bio16, Bio19, HI, slope	Bio7 (45.60%); Bio16 (12.30%); Bio6 (11.24%); Bio19 (8.81%)
Troides aeacus	*Aristolochia liukiuensis*	Aspect, Bio03, Bio07, Bio09, Bio12, Bio15, elevation, HI, slope	Bio7 (26.71%); Bio12 (20.98%); Elevation

			(12.46%); Bio9 (11.42%)
Troides aeacus	*Aristolochia griffithii*	Aspect, Bio02, Bio03, Bio04, Bio06, Bio15, Bio17, Bio18, elevation, HI, slope	Bio4 (29.00%); Bio3 (14.24%); Elevation (12.55%); Bio6 (10.18%)
