# Supplementary material for: Integrating Host Plant Availability into Ensemble Models Reveals Divergent Responses of Three Endangered Papilionid Butterflies to Climate Change in China
Source: Biology (Basel). 2026 Jul 24;15(15):1232. doi: 10.3390/biology15151232 (PMC13465108; doi:10.3390/biology15151232)
Supplement: Supplementary file 1 [file biology-15-01232-s001.zip › Table S5. Model performances of larval host plants of the three papilionid butterflies in China..pdf]

Table S5. Model performances of larval host plants of the three papilionid butterflies in China.

Butterfly species	Host plant	Occurrence record	Single models in ensemble model	Number of single models retained	Ensemble model AUC (mean ± SD)	Ensemble model TSS (mean ± SD)
Luehdorfia chinensis	*Asarum sieboldii*	161	GLM; GBM; CTA; FDA; MARS; MAXENT	6	0.915 ± 0.016	0.716 ± 0.044
Luehdorfia chinensis	*Asarum forbesii*	155	GLM; GBM; GAM; CTA; ANN; FDA; MARS; RF; MAXENT	9	0.970 ± 0.006	0.860 ± 0.021
Teinopalpus imperialis	*Daphne bholua*	27	GLM; GAM; CTA; ANN; FDA; MAXENT	6	0.976 ± 0.014	0.907 ± 0.058
Teinopalpus imperialis	*Yulania campbellii*	39	GLM; GAM; CTA; FDA	4	0.983 ± 0.007	0.951 ± 0.018
Troides aeacus	*Aristolochia debilis*	337	GLM; GBM; CTA; ANN; FDA; MARS; MAXENT	7	0.916 ± 0.015	0.725 ± 0.038
Troides aeacus	*Aristolochia kaempferi*	76	GLM; GAM; FDA; MARS	4	0.946 ± 0.019	0.821 ± 0.069
Troides aeacus	*Aristolochia zollingeriana*	32	GLM; GBM; CTA; ANN; FDA; MARS; MAXENT	7	0.936 ± 0.053	0.873 ± 0.089
Troides aeacus	*Aristolochia cucurbitifolia*	56	GLM; GBM; GAM; CTA; ANN; FDA; MARS; RF; MAXENT	9	0.978 ± 0.014	0.955 ± 0.025
Troides aeacus	*Aristolochia liukiuensis*	10	GBM; CTA; ANN; FDA; MAXENT	5	0.944 ± 0.156	0.847 ± 0.242
Troides aeacus	*Aristolochia griffithii*	21	GLM; GAM; CTA; ANN; FDA; MARS; MAXENT	7	0.972 ± 0.016	0.952 ± 0.021
