# Supplementary material for: Integrating Host Plant Availability into Ensemble Models Reveals Divergent Responses of Three Endangered Papilionid Butterflies to Climate Change in China
Source: Biology (Basel). 2026 Jul 24;15(15):1232. doi: 10.3390/biology15151232 (PMC13465108; doi:10.3390/biology15151232)
Supplement: Supplementary file 1 [file biology-15-01232-s001.zip › Table S6. Changes in landscape pattern indices of three papilionid butterflies in China after incorporating host availability under different climate sce.pdf]

Table S6. Changes in landscape pattern indices of three papilionid butterflies in China after incorporating host availability under different climate scenarios.

Species	Climate scenario	NP			MPA (km²)			LPI (%)			SPLIT		
		Env-only	Env+Host	ΔNP (%)	Env-only	Env+Host	ΔMPA (%)	Env-only	Env+Host	ΔLPI (%)	Env-only	Env+Host	ΔSPLIT (%)
Luehdorfia chinensis	Current	9931	5642	-43.19	8.15	2.44	-70.18	24.42	12.71	-47.95	16.77	61.86	268.87
	2050-SSP126	3347	6640	98.39	25.14	2.05	-91.85	25.38	11.93	-53.03	15.52	70.21	352.38
	2050-SSP245	2953	6918	134.27	29.51	2.02	-93.15	26.39	13.01	-50.74	14.36	59.08	311.49
	2050-SSP585	7932	3536	-55.42	10.10	4.05	-59.90	24.00	13.48	-43.83	17.37	55.01	216.70
	2070-SSP126	6432	5370	-16.51	12.88	2.63	-79.58	24.82	13.59	-45.21	16.23	54.10	233.27
	2070-SSP245	5187	7511	44.80	15.79	2.00	-87.33	24.58	13.24	-46.14	16.55	56.98	244.29
	2070-SSP585	6378	6995	9.67	12.89	2.24	-82.62	24.99	14.39	-42.38	16.02	48.15	200.56
	2090-SSP126	4303	8545	98.58	19.39	1.80	-90.72	25.28	13.37	-47.11	15.65	55.87	257.00
	2090-SSP245	7052	5025	-28.74	11.16	2.54	-77.24	23.49	12.33	-47.51	18.12	65.77	262.97
	2090-SSP585	5112	6474	26.64	16.03	2.56	-84.03	24.88	15.12	-39.27	16.15	43.68	170.46

	Mean	5410.67	6334.89	17.08	16.99	2.43	-85.70	24.87	13.38	-46.16	16.22	56.54	248.58
Teinopalpus imperialis	Current	70	11631	16515.71	1356.11	0.71	-99.95	29.01	5.26	-81.87	11.88	353.38	2874.58
	2050-SSP126	652	3967	508.44	142.19	0.79	-99.44	28.28	2.34	-91.73	12.50	1792.64	14241.12
	2050-SSP245	3378	4455	31.88	25.41	2.76	-89.18	26.00	11.74	-54.85	14.79	72.51	390.26
	2050-SSP585	879	3898	343.46	106.15	0.81	-99.24	28.49	1.57	-94.52	12.32	3531.04	28561.04
	2070-SSP126	2042	6103	198.87	44.62	1.07	-97.58	27.78	5.77	-79.19	12.96	299.69	2212.50
	2070-SSP245	70	661	844.29	1355.50	16.83	-98.76	29.00	10.85	-62.59	11.89	84.82	613.37
	2070-SSP585	895	4507	403.58	102.04	0.73	-99.28	27.87	2.23	-92.00	12.87	1899.02	14655.32
	2090-SSP126	2237	3149	40.77	40.37	1.01	-97.50	27.09	1.96	-92.76	13.62	2457.94	17946.55
	2090-SSP245	334	3357	905.09	282.50	1.00	-99.64	28.83	2.41	-91.64	12.03	1691.85	13963.59
	2090-SSP585	1259	3112	147.18	72.20	1.03	-98.57	27.73	1.57	-94.37	13.00	3562.18	27301.38
	Mean	1305.11	3689.89	182.73	241.22	2.89	-98.80	27.90	4.49	-83.91	12.89	1710.19	13167.57
Troides aeacus	Current	5088	3858	-24.17	15.86	6.79	-57.19	23.50	25.29	7.62	18.09	15.61	-13.71
	2050-SSP126	7709	1863	-75.83	10.70	14.54	35.89	24.53	26.77	9.13	16.62	13.94	-16.13

2050-SSP245	8603	1370	-84.08	9.64	19.60	103.32	24.76	26.32	6.26	16.31	14.42	-11.59
2050-SSP585	6546	1604	-75.50	12.74	16.98	33.28	25.03	26.65	6.47	15.96	14.06	-11.90
2070-SSP126	6382	2636	-58.70	13.09	10.13	-22.61	25.20	27.10	7.54	15.74	13.61	-13.53
2070-SSP245	8145	2868	-64.79	10.13	9.54	-5.82	24.83	27.60	11.16	16.22	13.12	-19.11
2070-SSP585	5735	2175	-62.07	14.37	12.22	-14.89	24.73	25.84	4.45	16.34	14.96	-8.51
2090-SSP126	4385	2090	-52.34	19.04	12.49	-34.40	25.21	25.49	1.11	15.73	15.37	-2.35
2090-SSP245	7115	2064	-70.99	11.65	12.86	10.47	24.94	26.77	7.34	16.08	13.95	-13.18
2090-SSP585	8397	1722	-79.49	9.73	15.79	62.18	24.54	26.54	8.15	16.60	14.18	-14.64
Mean	7001.89	2043.56	-70.81	12.34	13.80	11.75	24.86	26.56	6.84	16.18	14.18	-12.36

Note: NP = number of patches; MPA = mean patch area; LPI = largest patch index; SPLIT = splitting index. “Env-only” refers to models constructed using only environmental variables, whereas “Env+Host” refers to models incorporating both environmental variables and host plant availability. ΔNP, ΔMPA, ΔLPI and ΔSPLIT indicate the percentage of landscape pattern index from Env-only to Env+Host, respectively. Mean indicates the average across future climate scenarios.
