# Supplementary material for: Integrating Host Plant Availability into Ensemble Models Reveals Divergent Responses of Three Endangered Papilionid Butterflies to Climate Change in China
Source: Biology (Basel). 2026 Jul 24;15(15):1232. doi: 10.3390/biology15151232 (PMC13465108; doi:10.3390/biology15151232)
Supplement: Supplementary file 1 [file biology-15-01232-s001.zip › Table S7. MESS-based extrapolation-risk cell percentages (%) for future suitable habitat projections of three papilionid butterflies in China..pdf]

Table S7. MESS-based extrapolation-risk cell percentages (%) for future suitable habitat projections of three papilionid butterflies in China.

Future scenario	*Luehdorfia chinensis*		*Teinopalpus imperialis*		*Troides aeacus*	
	Env-only	Env+Host	Env-only	Env+Host	Env-only	Env+Host
2050-SSP126	0.84	0.35	0.78	0.40	0.77	0.35
2050-SSP245	1.55	0.93	1.66	1.00	0.87	0.41
2050-SSP585	1.13	0.54	1.12	0.64	1.08	0.57
2070-SSP126	0.74	0.39	0.89	0.51	0.93	0.43
2070-SSP245	1.74	1.25	2.01	1.40	1.18	0.66
2070-SSP585	1.49	1.02	1.86	1.42	1.73	1.09
2090-SSP126	1.50	0.88	1.70	0.92	1.47	0.81
2090-SSP245	2.17	1.59	2.41	1.77	1.42	0.75
2090-SSP585	2.11	1.03	9.64	10.7	1.83	1.15

Note: Values represent the percentage (%) of valid projection grid cells with negative Multivariate Environmental Similarity Surface (MESS) values. Negative MESS values indicate environmental conditions that are dissimilar to, or outside, the environmental space represented by the calibration data.
